# Role of the Extremolytes Ectoine and Hydroxyectoine as Stress Protectants and Nutrients: Genetics, Phylogenomics, Biochemistry, and Structural Analysis

**DOI:** 10.3390/genes9040177

**Published:** 2018-03-22

**Authors:** Laura Czech, Lucas Hermann, Nadine Stöveken, Alexandra A. Richter, Astrid Höppner, Sander H. J. Smits, Johann Heider, Erhard Bremer

**Affiliations:** 1 Laboratory for Microbiology, Department of Biology, Philipps-University Marburg, Karl-von-Frisch Str. 8, D-35043 Marburg, Germany; lauraczech@hotmail.de (L.C.); lucas.hermann@biologie.uni-marburg.de (L.H.); nadine@stoeveken.com (N.S.); alexandra.richter@biologie.uni-marburg.de (A.A.R.); heider@staff.uni-marburg.de (J.H.); 2 LOEWE—Center for Synthetic Microbiology, Philipps-University Marburg, Hans-Meerwein Str. 6, D-35043 Marburg, Germany; 3 Center for Structural Studies, Heinrich-Heine University Düsseldorf, Universitäts Str. 1, D-40225 Düsseldorf, Germany; astrid.hoeppner@uni-duesseldorf.de (A.H.); sander.smits@hhu.de (S.H.J.S.); 4 Institute of Biochemistry, Heinrich-Heine University Düsseldorf, Universitäts Str. 1, D-40225 Düsseldorf, Germany

**Keywords:** osmotic stress, high salinity, growth temperature extremes, enzymes, crystal structures, gene expression, genomics, chemical chaperones, biotechnology

## Abstract

Fluctuations in environmental osmolarity are ubiquitous stress factors in many natural habitats of microorganisms, as they inevitably trigger osmotically instigated fluxes of water across the semi-permeable cytoplasmic membrane. Under hyperosmotic conditions, many microorganisms fend off the detrimental effects of water efflux and the ensuing dehydration of the cytoplasm and drop in turgor through the accumulation of a restricted class of organic osmolytes, the compatible solutes. Ectoine and its derivative 5-hydroxyectoine are prominent members of these compounds and are synthesized widely by members of the Bacteria and a few Archaea and Eukarya in response to high salinity/osmolarity and/or growth temperature extremes. Ectoines have excellent function-preserving properties, attributes that have led to their description as chemical chaperones and fostered the development of an industrial-scale biotechnological production process for their exploitation in biotechnology, skin care, and medicine. We review, here, the current knowledge on the biochemistry of the ectoine/hydroxyectoine biosynthetic enzymes and the available crystal structures of some of them, explore the genetics of the underlying biosynthetic genes and their transcriptional regulation, and present an extensive phylogenomic analysis of the ectoine/hydroxyectoine biosynthetic genes. In addition, we address the biochemistry, phylogenomics, and genetic regulation for the alternative use of ectoines as nutrients.

## 1. Introduction

Microorganisms face myriad stressful conditions and nutrient limitations in their natural habitats; challenging circumstances to which they must react in a timely manner to ensure survival, persistence, and growth. An important parameter that affects essentially all microorganisms is the osmolarity/salinity of their surroundings [1,2,3,4,5,6], as increases or decreases in the environmental water activity will inevitably trigger water fluxes across the cytoplasmic membrane. 

Water is the active matrix of life [7,8], and the invention of the semi-permeable cytoplasmic membrane was a key event in the evolution of primordial cells. This membrane provided a confined space for the faithful copying of the genetic material, a reaction vessel for biochemical transformations and for the generation of energy to fuel growth. The cytoplasm of microorganisms is a highly crowded compartment caused by large concentrations of nucleic acids, proteins, and metabolites [9]. Together, these compounds generate a considerable osmotic potential [10] and thereby instigate osmotically driven water influx, a process that in turn causes the build-up of a hydrostatic pressure in walled cells, the turgor [2,10,11,12,13,14]. Turgor is considered essential for cell growth in many bacteria [15]. As microbial cells seem to strive to attain crowding homeostasis [9], they maintain turgor within physiologically acceptable boundaries through the accumulation and expulsion of ions and organic solutes [1,2,3,4,5,6], and they accomplish this even when faced with sudden fluctuations in the external osmolarity, or when they are exposed to persistent high or low osmolarity surroundings.

The development of the cytoplasmic membrane was a prerequisite for the evolution of microbial cells as we know them today; however, its semi-permeable nature makes cells vulnerable to osmotic fluctuations in their surroundings [2,6,10,11]. In extreme cases, the integrity of the cell is threatened by excessive water influx and a concomitant build-up of turgor to non-sustainable levels (under hypo-osmotic conditions) [16,17,18,19,20], or the ability of the cell to perform vital physiological tasks is impaired by the dehydration of the cytoplasm and the ensuing reduction/collapse of turgor when water exits the cell (under hyperosmotic conditions) [2,10]. It is apparent that coordinated cellular stress responses are needed to prevent such catastrophic effects.

Despite the existence of aquaporins in microorganisms that mediate diffusion-driven accelerated water fluxes across the cytoplasmic membrane [21,22], no microorganism can actively pump water (by means of an energy consuming process) into or out of the cell to compensate for water fluxes through this membrane that are instigated by changes in the external osmolarity. Microorganisms can, however, actively influence the direction and scale of water fluxes into or out of the cell by dynamically modulating the osmotic potential of the cytoplasm through the accumulation or expulsion of ions and organic compounds [2,5,10,11,20]. These combined activities allow microbial cells to cope dynamically with increases and decreases in the external osmolarity and are also crucial for their ability to colonize habitats with permanently high salinities/osmolarities [23,24]. 

**Figure 1:**
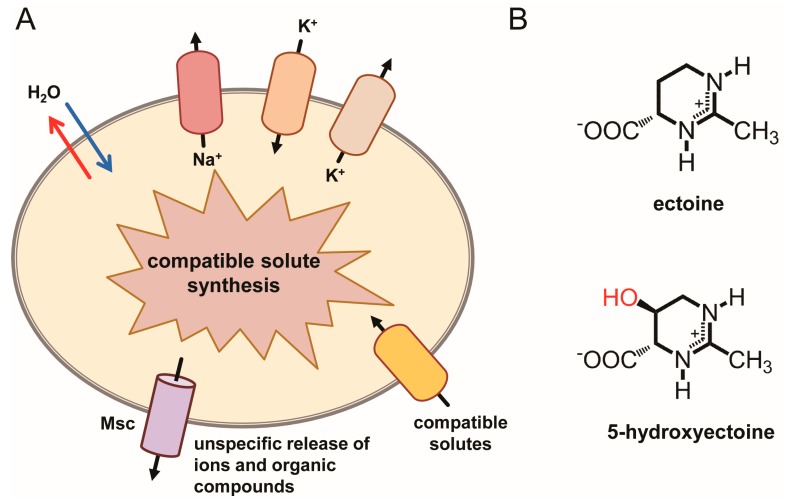
(**A**) General overview of the microbial *salt-out* osmostress adaptation strategy. The components, ion fluxes, and compatible solute pools generated via import and synthesis under hyperosmotic conditions [1,2], and the non-specific release of ions and low molecular weight organic compounds via mechanosensitive channels (Msc) under suddenly imposed hypo-osmotic circumstances are depicted [11,20]. (**B**) Chemical structures of the compatible solutes ectoine and 5-hydroxyectoine.

When exposed to high-osmolarity environments, microorganisms amass ions and organic osmolytes to increase the osmotic potential of their cytoplasm (Figure 1A). This curbs water efflux and promotes water influx, thereby balancing the vital osmotic gradient across the cytoplasmic membrane under osmotically unfavorable environmental circumstances [4,5,24,25]. An increase in the osmotic potential of the cytoplasm can be accomplished by one of two cellular adjustment strategies. These are to accumulate high levels of either selected salt ions (primarily K^+^ and Cl^−^) (the *salt-in* strategy) or of physiologically compliant organic osmolytes, the compatible solutes (the *salt-out* strategy) [1,5,25]. 

While the accumulation of ions and/or organic osmolytes ensures survival and growth of microorganisms under high osmolarity/salinity conditions (Figure 1A), the high intracellular pools of the very same compounds threatens the integrity of the cell when it is suddenly exposed to a drop in the external osmolarity [11,16,17,18,20]. Such conditions occur, for instance, for soil-dwelling bacteria upon rainfall and by washout into freshwater sources, for microorganisms living in brackish ecosystems, and for enteric bacteria when they exit the intestine of their host. The ensuing osmotic down-shocks require a very rapid cellular adjustment response in order to avoid bursting [11,20,26,27]. For instance, turgor pressure in *Escherichia coli* has been estimated to lie between 0.3 atm and 3 atm [13,14], values that increase practically instantaneously to about 20 atm upon a sudden and severe osmotic down-shift [11]. Such a drastic increase in turgor cannot be restrained by the stress-bearing peptidoglycan sacculus [28,29] of the cell wall alone, and consequently, the cell would burst [11,16,17,18,19]. 

To avoid rupture under suddenly imposed hypo-osmotic condition, bacteria engage safety valves embedded in the cytoplasmic membrane, the mechanosensitive channels (Figure 1A). An immediate consequence of the osmotically driven water influx upon down-shock is the gating of these channels, a process caused by the increase in the tension of the lateral plain of the cytoplasmic membrane as the consequence of increased turgor. Often, multiple types of mechanosensitive channels [MscM (mini), MscS (small), MscL (large)] are present in a given microbial cell and they possess different pore sizes and gating behaviors [11,20,26,27,30,31,32]. Their transient opening allows a rapid, non-specific jettison of low-molecular-weight solutes (both ions and organic compounds), whereupon the mechanosensitive channels close again as a result of the reduction in the osmotic potential of the cytoplasm and the ensuring decrease in turgor. Consequently, by relying on the turgor-driven opening and closing of mechanosensitive channels (Figure 1A), the cell can mount a timely and graded response to the severity of the suddenly imposed osmotic imbalance [11,20,30,32]. Mechanosensitive channels are essential for cellular survival under severe osmotic down-shock conditions [11,16,17,18], but not during steady-state growth at either high or low osmolarity [16,17]. 

## 2. The *Salt-In* and *Salt-Out* Strategies for Coping with High Osmolarity Environments

The *salt-in* strategy relies on the massive accumulation of K^+^ and Cl^−^ ions from environmental sources through transport and the active extrusion of cytotoxic Na^+^ ions from the cell [24,25]. As a consequence of the permanently high ion content of the cytoplasm, the biochemical properties and the compositions of all proteins have to be adjusted to keep them soluble and functional. On an evolutionary time scale, this has left an acidic signature on the proteome with a narrow distribution of isoelectric points as the consequence of reduced hydrophobicity of proteins and a strong increase in negatively charged amino acids exposed on protein surfaces [33,34,35]. The *salt-in* strategy is energetically favorable [36,37], and is thus particularly effective in habitats with sustained very high salinity [23,24,25], but seems less useful in environments in which the salinity/osmolarity fluctuates more often [1,3,4,5]. 

A more flexible adjustment to high-osmolarity environments is provided by the *salt-out* strategy, which is therefore used widely in the microbial world [1,5]. This strategy also entails a rapid uptake of potassium ions as an emergency reaction to a sudden challenge by high osmolarity, but part of the initially amassed K^+^ pool is subsequently replaced by the cells through types of organic osmolytes that are highly compliant with cellular functions, the compatible solutes (Figure 1A) [1,2,3,4]. In this way, the cell attains a level of hydration of the cytoplasm that is appropriate for biochemical processes and simultaneously upholds turgor without concurrently raising the intracellular ionic strength, as this would greatly impair most physiological activities of the cell [2,10]. As an added benefit, the *salt-out* strategy does not require an evolutionary adjustment in the proteome profile. However, the amassing of compatible solutes, either through uptake or synthesis [1,2], is energetically substantially more demanding than the *salt-in* strategy [36,37]. 

It was thought that the *salt-in* and *salt-out* strategies were mutually exclusive, and that the observation of an acidic proteome was predictive for the use of the *salt-in* strategy. While this is probably correct in general, recent findings require a modification of this long-held view [38,39]. For instance, a group of *Halobacteriales*, halophilic Archaea, was found to combine a high K^+^ cytoplasm with the accumulation of the compatible solutes trehalose and 2-sulfotrehalose [38]. Notably, in the extreme halophilic archaeon *Halobacterium salinarum*, even chemotaxis towards the osmoprotectants glycine betaine, carnitine, and choline has been detected [40]. While pathways for the synthesis of compatible solutes in extremely halophilic Archaea seem to be rare, those for the uptake of compatible solutes are prevalent [41]. These observations indicate that some haloarchaea, at least under certain environmental conditions, might combine the *salt-in* and *salt-out* strategies to combat the detrimental effects of high salinity on cellular physiology. It is also noteworthy that in several phylogenetically closely related members of the genus *Halorhodospira*, different osmostress adaptation strategies can be found. For instance, in *Halorhodospira halophila*, a highly acidic proteome is combined with a high K^+^/Cl^−^ pool, while in *Halorhodospira halochloris* this is not the case; instead, this halo-alkaliphilic microorganism is a producer of compatible solutes [24,39,42]. Finally, it was thought that obligate protein halophilicity was the price evolution had to pay for the *salt-in* strategy, but *H. halophila* can substantially reduce its K^+^ content from about 2.1 M in cells grown at high salinity to a level (0.4 M) comparable to that of *E. coli* when it is cultivated in media with more moderate salt concentrations (0.21 M) [39]. As stated by A. Oren in his insightful review on intracellular K^+^ and acid proteomes [24], the previously clear-cut picture of a correlation between phylogenetic affiliation and mode of salt adaptation, and the correlation between acidic proteomes, accumulation of high K^+^ content, and the use of compatible solutes needs a careful re-evaluation. The findings that some microorganisms combine an acidic proteome with the accumulation of compatible solutes [24,38,41,43], and that a substantial reduction in K^+^ content can be accomplished in *salt-in* adopters under more moderate salt-stress conditions [39], prompts the exploration of new avenues of research and raises intriguing questions about the role played by protein halophilicity in the evolution of microbial osmostress responses. 

## 3. Compatible Solutes 

### Stress-Relieving Cytoprotectants Used in All Three Domains of Life

One of the main physiological roles played by compatible solutes in Archaea, Bacteria and Eukarya is to counteract the negative effects of high external osmolarity on cellular hydration and volume [1,5,44,45,46,47]. They are therefore amassed by microbial cells with pool sizes that increase in accordance with the degree of the imposed osmotic stress. Compatible solutes are operationally defined as organic osmolytes that can be accumulated by cells to exceedingly high levels without disturbing vital cellular functions [48]. They are also addressed as counteracting [44] or compensatory [49] organic solutes to highlight their cytoprotective effects against challenges in addition to those posed by high osmolarity/salinity; for example, low and high temperate extremes, hydrostatic pressure, freezing, desiccation, and the denaturation of macromolecules by ions and urea. These types of solutes have physico-chemical properties that distinguish them from other types of organic compounds, and similar types of low-molecular weight compounds have been selected during the course of evolution in all three domains of life to fulfill cellular functions as cytoprotectants [1,5,45,46,47,50]. One of the most widely distributed compatible solutes on Earth is glycine betaine. This compound, and many other compatible solutes as well, is not only employed as an effective osmostress protectant, but also also provides cytoprotection against challenges posed by extremes in growth temperature and hydrostatic pressure, attributes that lead to the description of particular compatible solutes as thermolytes and piezolytes, respectively [50,51,52,53,54,55,56,57,58].

A hallmark of compatible solutes is their preferential exclusion from the immediate hydration shell of proteins [59], an effect largely caused by unfavorable interactions between these solutes and the protein backbone [60,61,62]. This preferential exclusion [59] leads to an uneven distribution of compatible solutes in the cell water and therefore generates a thermodynamic driving force that acts against the denatured and aggregated state of proteins. Hence, proteins are forced to adopt a compact and well-folded state under intracellular unfavorable osmotic and ionic conditions to minimize the number of excluded compatible solute molecules from surfaces [61,62]. Consequently, the accumulation of compatible solutes not only has beneficial effects on cellular hydration and maintenance of turgor, but also promotes the functionality of macromolecules (e.g., in particular, proteins and membranes, and protein:DNA interactions) under otherwise activity-inhibiting conditions [54,63,64,65,66,67,68,69]. 

The function-preserving property of compatible solutes has attracted considerable biotechnological interest, and the term “chemical chaperones” was coined in the literature [54,70,71] to reflect the beneficial effects of these compounds as protein stabilizers and protectants for entire cells [72,73,74,75]. The function of compatible solutes as chemical chaperones will certainly contribute to their role as protectants against extremes in either high or low temperatures for microorganisms [51,52,53,54,76,77,78,79,80,81], an underappreciated physiologically important attribute of these types of solutes. For instance, the hyperthermophile *Archaeoglobus fulgidus* cannot grow at 90 °C in a chemically fully defined minimal medium [82], despite the fact that this archaeon synthesizes the extremolyte diglycerol phosphate in response to heat stress, an excellent stabilizer of protein function at high temperature [56,83]. However, the addition of 1 mM glycine betaine to the growth medium and its import via the heat stress inducible ProU ABC transporter efficiently rescued growth of *A. fulgidus* at the extreme temperature of 90 °C [82]. In other words, glycine betaine can act as an effective thermoprotectant for a hyperthermophile. Similarly, a defect in the molecular chaperone DnaK that causes thermo-sensitivity of *E. coli* at 42 °C, can be functionally rescued by an external supply of the compatible solutes l-proline, glycine betaine and by the glycine betaine biosynthetic precursor choline [53,76]. Furthermore, a broad spectrum of compatible solutes serves as thermoprotectants at the cutting upper (about 52 °C) and lower (about 13 °C) temperature boundaries for growth of *Bacillus subtilis* in a chemically defined minimal medium [51,52,84,85]. 

Although originally coined for unusual compatible solutes produced by microorganisms that live in habitats with extreme temperature, salt and pH profiles [86], the term extremolyte can be applied to these types of solutes in general [5,55,57,86,87]. This is exemplified by the above-cited example of the impressive thermoprotection of *A. fulgidus* by the “ordinary” compatible solute glycine betaine. Within the domain of the Bacteria, important representatives of compatible solutes are the amino acid l-proline, the trimethylammonium compound glycine betaine and its analogue arsenobetaine, proline-betaine, the sugar trehalose, the heteroside glucosylglycerol, the sulfur-containing dimethylsulfoniopropionate (DMSP), and the tetrahydropyrimidines ectoine and 5-hydroxyectoine. We refer readers to several excellent overviews that address the diversity of compatible solutes produced and imported by Bacteria and Archaea [4,5,55,57,87]. 

Here we focus on the synthesis and import of the compatible solute ectoine and its derivative 5-hydroxyectoine (Figure 1B), their stress-relieving properties, and their alternative function as versatile microbial nutrients. 

## 4. Ectoine and Hydroxyectoine

### 4.1. Discovery 

Ectoine [(4*S*)-2-methyl-1,4,5,6-tetrahydropyrimidine-4-carboxylic acid] (Figure 1B) was originally discovered in the extremely halophilic phototrophic purple sulfur bacterium *Ectothiorhodospira halochloris* (now taxonomically re-classified as *H. halochloris*) by Galinski et al., in 1985 [88]. This seminal discovery was followed by the detection of a hydroxylated derivative of ectoine, 5-hydroxyectoine [(4*S*,5*S*)-2-methyl-5-hydroxy-1,4,5,6-tetrahydropyrimidine-4-carboxylic acid] by Inbar and Lapidot in 1988 in the Gram-positive soil bacterium *Streptomyces parvulus* [89], a compound that the authors initially referred to as THP (A) [2-methyl-4-carboxy-5-hydroxy-3,4,5,6-tetrahydropyrimidine]. Ectoine and 5-hydroxyectoine (Figure 1B) can chemically be classified as either heterocyclic amino acids or as partially hydrogenated pyrimidine derivatives [88,89,90]. Both ectoine and 5-hydroxyectoine were initially viewed as rare naturally occurring compatible solutes (e.g., in comparison with the almost universally distributed glycine betaine molecule). However, improved screening procedures using HPLC analysis and, in particular, ^13^C-natural abundance NMR spectroscopy revealed their widespread synthesis in bacteria in response to high salinity [25,55]. Ectoine producers can be found within a physiologically and taxonomically diverse set of microbial species [91,92,93,94]. Today, ectoines are known to be one of the most ubiquitously distributed compatible solutes in the microbial world.

The identification of ectoine biosynthetic genes (*ectABC*) [95] and of the gene coding for the ectoine hydroxylase (*ectD*) [96,97,98] proved to be a major step forward for an in silico assessment of the distribution of ectoine/5-hydroxyectoine biosynthesis in microorganisms, an approach made possible by the rapid and unabated growth in the number of available genome sequences of Bacteria and Archaea [92,93]. Producers of ectoines are primarily found among members of the domain of the Bacteria [91,93,99] and in a rather restricted number of the Archaea [92]. Surprisingly, ectoine/5-hydroxyectoine biosynthetic genes and production of ectoine have recently also been detected in some bacteriovorus unicellular Eukarya [100,101,102] that live in permanently high-salinity ecosystems [103]; these protists probably acquired the ectoine/5-hydroxyectoine biosynthetic genes through lateral gene transfer from their food bacteria [100,104]. 

### 4.2. Physico-Chemical Attributes

Like other compatible solutes [61,62], ectoine and 5-hydroxyectoine (Figure 1B) are low-molecular mass compounds that are highly soluble in water (about 4 M at 20 °C) [105], thereby allowing the amassing of these compounds to near molar concentrations in severely osmotically stressed microbial cells [91,94,106]. A variety of biophysical techniques have been used to study the effects of ectoine on the hydration of proteins and cell membranes and on interactions mediated via hydrogen bonding. Collectively, these data showed that ectoine is excluded from the monolayer of dense hydration water around soluble proteins and from the immediate hydration layer at the membrane/liquid interface [66,105]. Ectoine enhances the properties of hydrogen bonds in aqueous solutions and thereby contributes to the dynamics and stabilization of macromolecular structures. Ectoine possesses a negatively charged carboxylate group attached to a ring structure that contains a delocalized positive charge (Figure 1B). The resulting interplay between hydrophilic and hydrophobic forces influences water-water and water-solute interactions [107] and thereby exerts strong effects on the hydration of ectoine itself, the binding of ions and the influence on the local water structure [108,109,110,111]. 

Molecular dynamics simulations have indicated that ectoine and 5-hydroxyectoine are strong water-binders and are able to accumulate seven and nine water molecules, respectively, around them at a distance smaller than 0.6 nm [67]. This results in the formation of a large number of hydrogen bonds at specific functional groups of molecules. Furthermore, these studies indicated that the water-binding behavior of ectoines is not abrogated or perturbed at high salt concentrations [67]. The influence of ectoines on the local water structure also exerts pronounced effects on protein-DNA interactions [109,112,113,114], a crucial effect that might alter the transcriptional profile of salt-stressed cells on a genome-wide scale [68]. Collectively, the physico-chemical attributes of ectoines allow a physiologically adequate hydration of the cytoplasm upon their osmostress-responsive accumulation, afford effects on the local water structure, and also exert a major protective influence on the stability of proteins and the functionality of macromolecules [72,73,105,114,115]. 

### 4.3. Stress-Protective Properties 

Ectoine and 5-hydroxyectoine are produced by microorganisms in response to true osmotic stress, and not just in response to increases in the external salinity [116]. In cases where the build-up of ectoine/5-hydroxyectoine pools has been studied in more detail, there is often a linear relationship between the cellular content of these solutes and the external salinity/osmolarity [116,117,118]. This finding implies that bacterial cells can perceive incremental increases in the degree of the environmentally imposed osmotic stress, can process this information genetically/physiologically, and can then set its ectoine/5-hydroxyectoine biosynthetic capacity in a finely tuned fashion to relieve the constraints imposed by high-osmolarity on cellular hydration, physiology, and growth [1,2,3,4]. As described in greater detail in Section 5.2, high-osmolarity-dictated increases in the cellular ectoine pools are largely accomplished through osmotically-responsive increases in the transcription of the ectoine/5-hydroxyectoine biosynthetic genes, although there might be post-transcriptional effects as well. Attesting to the role of ectoine as a potent osmostress protectant is the finding that the disruption of the *ectABC* biosynthetic genes (see Section 5.1) causes osmotic sensitivity [119,120] and the genetic disruption of the gene (*ectD*) for the ectoine hydroxylase in *Chromohalobacter salexigens* impairs the ability to cope effectively with high growth temperature extremes [97]. 

In addition, environmental challenges other than high osmolarity also trigger enhanced production of ectoines in some microorganisms, in particular, extremes in either high or low growth temperatures [77,81,97]. Furthermore, the function of ectoines as thermolytes is also manifested when microbial cells acquire these solutes from environmental sources through transport processes [78,79,121]. Although the term chemical chaperone is suggestive of a description of the function-preserving attributes of compatible solutes, it is not truly clear how the thermoprotective effects of ectoines are achieved on a biochemical and molecular level. We find it also important to note in this context that the mechanisms underlying the cytoprotective effects of ectoines at high and low temperature do not necessarily need to be the same. In addition, both processes might be, in their core, different from the cytoprotective effects exerted by ectoines when they act as osmostress protectants. 

In microorganisms that are capable of synthesizing both ectoine and 5-hydroxyectoine, a mixture of these two solutes is frequently found. Interestingly, such a 1:1 mixture (0.5 mM each) provided the best salt and heat stress protection to *Streptomyces coelicolor* when it was added to the growth medium [79]. However, there are also microorganisms that seem to produce almost exclusively 5-hydroxyectoine during osmotic stress and different growth phases of the culture [122,123]. 

An interesting phenomenon that has been dubbed osmolyte switching [124,125], plays an important role in the temporal dynamics of ectoine production in some microorganisms. For instance, *Halobacillus halophilus*, which uses a hybrid osmostress adjustment strategy of Cl^−^ and compatible solute accumulation [125], initially uses l-glutamate as its primary organic osmolyte and then switches to the synthesis of l-proline when the external salinity is further increased. A second switch in the preferred compatible solute then occurs from l-proline to ectoine at the transition from exponential to stationary phase [124,125]. Similarly, *Virgibacillus pantothenticus* initially relies on the synthesis of l-proline when it is osmotically challenged by moderate increases in the external salinity, and then triggers enhanced ectoine production once the salinity of the growth medium is increased above 0.6 M NaCl [77]. Hence, in microorganisms that produce several organic osmolytes, there seems to be, at least in certain cases, a temporal hierarchy in the type(s) of the dominantly synthesized compatible solute(s). Apparently, when the environmental and cellular circumstances get particularly tough, ectoine is preferentially produced. This notion fits nicely with the results of a study in which the dominantly produced compatible solute(s) in a substantial number of Bacilli were assessed by natural abundance ^13^C-NMR-spectroscopy [98,117]. Three groups were detected: (i) those that synthesize exclusively l-glutamate, (ii) those that synthesize l-glutamate and l-proline, and (iii) those that synthesize both l-glutamate and ectoine. Some members of this latter group also produce 5-hydroxyectoine. Although not studied in detail, there seems to be a correlation between the type of compatible solute synthesized and the degree of the attained osmostress resistance, with ectoine/5-hydroxyectoine producers being the most salt-stress tolerant Bacilli [98,117]. Presumably, this phenomenon is related to the different physico-chemical attributes of l-glutamate, l-proline, and ectoine and the ensuing effectiveness by which they can than serve as compatible solutes [107,126,127,128]. 

As mentioned above, a substantial increase in 5-hydroxyectoine content occurs not only in response to osmotic challenges in some microorganisms, but also when cells enter stationary phase [121,129]. This observation implies that the hydroxylated derivative of ectoine possesses stress-relieving properties that will allow the cell to better cope with the multitude of challenges imposed by stationary phase [130,131]. This attribute might stem from the frequently observed superior function-preserving properties of 5-hydroxyectoine when tested either in vivo [79,97] or in vitro [72,73,74,75,114,132,133,134]. Fourier transform infrared and electron spin resonance studies revealed that 5-hydroxyectoine has a substantially greater glass-forming propensity than ectoine, a trait that stems from stronger intermolecular hydrogen-bonds with the OH group of 5-hydroxyectoine (Figure 1B) [132]. As a consequence of the strongly increased glass transition temperature (87 °C for 5-hydroxyectoine versus 47 °C for ectoine), 5-hydroxyectoine is an excellent desiccation protectant, a characteristic that not only allows the stabilization of individual biomolecules, but the protection of entire cells from anhydrobiotic-induced damage [74,75]. Biosynthesis and external application of 5-hydroxyectoine can thus be exploited for synthetic anhydrobiotic engineering [84,85,132]. In *C. salexigens*, 5-hydroxyectoine has also been found to be a better protectant than ectoine against oxidative stress caused by an excess supply of iron in the growth medium [135]. 

In *Alcalivorans borkumensis* SK2, a member of a widely distributed genus dominating oil spills worldwide, ectoine has been suggested to function as a piezolyte by protecting the cell against excess hydrostatic pressure [136]. However, a previous study found no evidence for such a function for ectoine by comparing the pressure survival of the piezo-sensitive *E. coli* cell (non-ectoine producer) with that of *C. salexigens* (an ectoine producer) [137]. Ectoines also have pronounced effects on the melting temperature of DNA, but ectoine and 5-hydroxyectoine differ in this regard. While ectoine lowers the melting temperature, 5-hydroxyectoine increases it [114]. Furthermore, ectoine protects DNA against the induction of single-strand breaks by ionizing radiation and serves as a scavenger for hydroxyl radicals [138,139,140]. Ectoine is also a potent protectant against UV-induced cellular stress [141,142]. Interesting stress-protective and function-preserving properties might also be derived from synthetic ectoines with reduced or expanded ring sizes [143] or by chemical modifications that provide a hydrophobic anchor (e.g., lauryl-ectoine) to the otherwise highly water-soluble ectoine molecule [144]. 

### 4.4. Biotechnological Production and Practical Applications of Ectoines

The excellent function-preserving attributes of ectoines have attracted considerable attention to their exploitation in the fields of biotechnology, skin care, and medicine [86,91,94,145,146,147]. This demand for ectoines for practical purposes has led to an industrial-scale production process that exploits *Halomonas elongata* as a natural and engineered cell factory, delivering ectoines on the scale of tons [86,91,94]. Data reported in the literature [148,149] estimate a worldwide production level of ectoines of about 15,000 tons per annum, which putatively have an estimated sales value of approximately 1000 US Dollars kg^−1^. However, another study reports a price for ectoine at between about 14,000 and 18,000 Euro kg^−1^ [150]. We are not certain what these numbers are actually based upon, since details pertinent to their calculations are not given in these publications [148,149,150]. However, there can be no doubt that ectoines are high-value natural products. By consulting catalog prices listed by vendors of laboratory chemicals (and not by the major industrial producer of ectoine; bitop AG, Dortmund, Germany; https://www.bitop.de/), the purchasing costs for one kg of ectoines ranges between 9000 Euro (Acadechem, Hong Kong, China) and 17,000 Euro (AppliChem, Darmstadt, Germany) kg^−1^ for ectoine, and the sale price for 1 kg of 5-hydroxyectoine is about 17,000 Euro (Merck, Darmstadt, Germany).

Of the extremolytes currently considered for practical applications [146], ectoine and 5-hydroxyectoine certainly have the greatest potential for sustained commercial exploitation [86,91,94,145,147]. It is outside the scope of this overview to address in depth the biotechnological production of ectoines in natural and synthetic microbial cell factories, or to describe in detail the varied practical applications for these compounds. Insightful reviews covering these topics have been published [86,91,94,145], and recent reports have summarized the current status of efforts to improve the productivity of natural and synthetic microbial cell factories for ectoines [116,123,151,152,153,154,155,156,157]. 

Briefly, the industrial-scale production scheme for ectoine relies on the highly salt-tolerant gammaproteobacterium *H. elongata* as a natural cell factory [86,158]. It exploits the massive production of ectoines under high-salinity growth conditions by this bacterium [158] and their non-specific release from the producer cells via the transient opening of mechanosensitive channels upon a severe osmotic down-shock [91,94,159]. Since the gating of mechanosensitive channels prevents cell rupture [11,20,32], the biomass formed during the originally high-cell density fermentation of *H. elongata* under osmotic stress conditions can be re-introduced into the fermentation vessel for a new round of ectoine production and release [91,94,159]. This innovative production process has been fashionably dubbed bacterial milking [159]. During subsequent strain development, the production process was amended by the use of *H. elongata* mutants that lack the TeaABC system, a TRAP-type [160] ectoine/5-hydroxyectoine-specific transporter that can serve as a recycling system for newly synthesized ectoines released, or actively excreted, from the *H. elongata* producer cells [161]. Use of *tea* mutants in the industrial production strain leads to the continuous accumulation of ectoines in the growth medium [94,161]. Ectoines jettisoned during osmotic downshifts of *H. elongata* cells, or released into the growth medium by the *tea* mutant strain, can be recovered from the fermentation medium with high yield and purity by down-stream processes via protein precipitation through acidification, cation exchange chromatography, and evaporation/crystallization [86,91,94,145,146,147]. 

A number of commercial applications for ectoines have been developed that rely, in their core, on the ability of ectoine and 5-hydroxyectoine to serve as water-attracting and water-structure-forming compounds [109,110,111], to stabilize macromolecules and entire cells through their chaperon and glass-forming effects [66,67,86,91,94,105,132,145], to protect DNA from ionizing radiation [138,140], and to prevent UV-induced cell damage of skin cells [141,142,145]. These latter two properties and the moisturizing effects of ectoines have fostered the development of a wide range of products for skin care and cosmetics [145]. Ectoines are used to stabilize enzyme activity in vitro, for promoting protein folding in vivo, for protecting molecules and cells against cycles of freezing and thawing, for promoting their desiccation resistance, for enhancing the resistance of cells and DNA against ionizing radiation and damage elicited by UV, as oxidative and temperature stress protectants, for preventing the impairment of cell membrane functions, for cytoprotection of eukaryotic cells and organs, and they have even been evaluated as protectants against neurodegenerative diseases [86,91,94,145,147]. 

Compatible solutes have also been explored as beneficial additives to biological waste and wastewater treatment systems to counteract osmotic and other types of environmental stresses [150]. In addition to glycine betaine and trehalose, the effects of ectoine have also been evaluated in this regard. A denitrifying microbial consortium has been used to study the effect of ectoine on denitrification at increased salinity. The addition of ectoine (1 mM) accelerated the de-nitrification process, promoted the almost complete removal of nitrates and nitrites relative to that of control samples in a shorter time frame, and enhanced the activity of key degradative enzymes [162]. The addition of ectoine also stimulated the Anammox process (by about 40%) under conditions of increased salinity [163]. While these pilot studies demonstrate the use of compatible solutes in general, and that of ectoine in particular, for these types of applications [150], it is unlikely that ectoine can ever be used in large-scale biological waste and wastewater treatment systems unless the production costs for ectoine would drop precipitously and become competitive with the bulk-chemical glycine betaine (196 Euro kg^−1^) (Merck, Darmstadt, Germany). 

## 5. Ectoine/5-Hydroxyectoine Biosynthetic Routes and Crystal Structures of Selected Enzymes

### 5.1. Biosynthetic Pathway: An Overview

**Figure 2:**
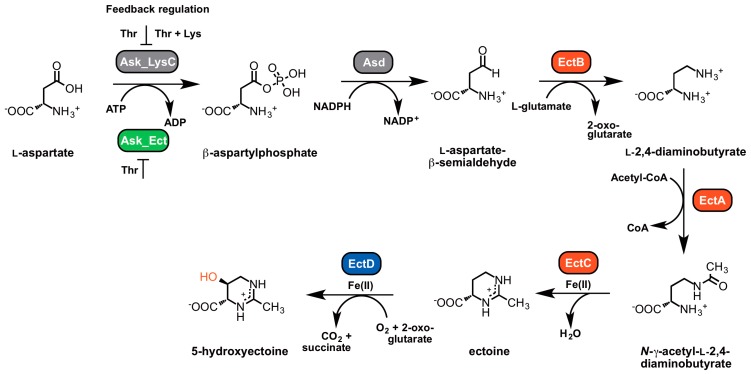
Routes for ectoine and 5-hydroxyectoine biosynthesis.

Three enzymes are involved in ectoine synthesis: l-2,4-diaminobutyrate (DABA) transaminase (EctB; EC 2.6.1.76), l-2,4-diaminobutyrate acetyltransferase (EctA; EC 2.3.1.178), and ectoine synthase (EctC; EC 4.2.1.108). 5-hydroxyectoine is formed in a subgroup of ectoine producers through a position- and stereo-specific hydroxylation of ectoine, an enzymatic reaction catalyzed by the ectoine hydroxylase (EctD; EC 1.14.11.55) (Figure 2). 

The ectoine biosynthetic route was originally elucidated by Peters et al. [164] through an analysis of enzyme activities present in cell-free extracts of *E. halochloris* and *H. elongata*. Ono et al. [165] subsequently made major contributions to an understanding of the biochemistry of the ectoine biosynthetic enzymes; these authors used purified EctABC proteins from *H. elongata* to study their enzymatic properties. In addition, biochemical procedures to study these enzymes from various methylotrophic bacteria were summarized by Reshetnikov et al. [166]. The biochemical properties of ectoine hydroxylase from *Salibacillus salexigens* were first determined by Bursy et al. [79,98], and Widderich et al. [92,93] subsequently studied this enzyme from a substantial number of Bacteria and from a single archaeon. Ectoine is synthesized from the precursor l-aspartate-β-semialdehyde (Figure 2), a central intermediate of microbial amino acid metabolism and cell wall and antibiotic synthesis [167]. In a sub-group of ectoine/5-hydroxyectoine producers, the ectoine/5-hydroxyectoine biosynthetic gene cluster contains a gene (*ask_ect*) for a specialized aspartokinase [93,168]. Its biochemical properties were studied by Stöveken et al. [122] using the Ask_Ect enzyme from *Pseudomonas stutzeri* A1501 and by Reshetnikov et al. [166] using the corresponding enzyme from *Methylobacterium extorquens* AM1.

In comparison with the energetic demands to sustain the *salt-in* osmostress adjustment strategy through the import of ions, implementation of the *salt-out* strategy through the production of massive amounts of compatible solutes is energetically very costly for microorganisms [36,37]. This is, of course, also true for the synthesis of ectoine. As calculated by A. Oren, the energy requirements (expressed in ATP equivalents) for the synthesis of a single ectoine molecule by an aerobic heterotroph growing on glucose corresponds to about 40 ATP equivalents and increases to approximately 55 ATP equivalents when ectoine is synthesized by an autotroph from CO_2_ [37]. These values closely resemble those calculated for the synthesis of the compatible solute glycine betaine under these two growth conditions [37] when it is produced either via the sequential methylation of glycine [169] or the through oxidation of choline [170,171,172]. From an energetic point of view, synthesis of ectoine and glycine betaine are considerable less expensive than that of the compatible solute trehalose, whose production by an aerobic heterotroph growing on glucose requires the expenditure of about 79 ATP equivalents, an energetic cost that rises to about 109 molecules of ATP when this disaccharide is produced by an autotroph from CO_2_ [37]. 

### 5.2. Characteristics of the Ectoine/5-Hydroxyectoine Biosynthetic Enzymes

#### 5.2.1. l-2,4-Diaminobutyrate Transaminase EctB

Ectoine synthesis starts with the transamination of the precursor l-aspartate-β-semialdehyde, a reaction catalyzed by the l-2,4-diaminobutyrate-2-oxoglutarate transaminase EctB. EctB might be a pyridoxal-5′-phosphate (PLP)-dependent enzyme [173] similar to other aminotransferases, and requires K^+^ for its activity and stability [165]. The EctB enzyme accepts l-aspartate-β-semialdehyde as its substrate and catalyzes the reversible transfer of an amino group from l-glutamate to the aldehyde group of the substrate, thereby forming l-2,4-diaminobutyrate (DABA) and 2-oxoglutarate (Figure 2). Biochemical characterization of EctB was reported for the orthologous enzymes from *H. elongata* [165] and *Methylomicrobium alcaliphilum* [99]. Both enzymes are homo-hexameric proteins and have a strong requirement of K^+^ for their enzymatic activity and stability. The preferred amino group donors are l-glutamate for the forward reaction (forming DABA) and DABA or 4-aminobutyrate for the reverse reaction (forming glutamate). Optimal catalytic activities were recorded for the enzyme from *H. elongata* at temperatures of 25 °C, a slightly alkaline pH of 8.6, and KCl concentrations of 0.5 M. Addition of NaCl (0.05–0.5 M) also enhanced the enzyme activity, but the enhancing effect of KCl in the range of 0.01–0.5 M on enzyme activity was much stronger. The apparent *K*_m_ values are 9.1 mM for the amino group donor l-glutamate and 4.5 mM for the amino group acceptor l-aspartate-β-semialdehyde [165]. 

An innovative approach was taken by Chen et al. [156] to identify variants of the *H. elongata* EctB enzyme with substantially enhanced catalytic activity. These authors re-engineered the AraC transcription factor from *E. coli* so that it would preferentially respond in its DNA-binding activity to the *ara* promoter (P_BAD_) to the cellular ectoine pool, instead of to its natural effector molecule l-arabinose. They then combined the synthetic AraC^Ect^ regulatory protein with a P_BAD_-ECFP fluorescent reporter system in a strain simultaneously expressing the *H. elongata ectABC* gene cluster on a plasmid. In this way, they were able to identify variants of the *ectABC* gene cluster, generating higher cellular ectoine pools. These strains carried amino acid substitutions in EctB, the enzyme that controls the flux of the precursor l-aspartate-β-semialdehyde into the ectoine biosynthetic route (Figure 2) [165,166]. One of the recovered *ectB* mutants simultaneously carried three mutations, leading to amino acid substitutions D180V/F320Y/Q325R. The encoded mutant EctB enzyme exhibited a notably improved (by about 4.1-fold) catalytic efficiency (*K*_cat_/*K*_m_), and thereby concomitantly increased cellular ectoine titers in the heterologous *E. coli* host by about 3.3-fold relative to a strain possessing the wild-type EctB protein [156]. The bio-sensing metabolic engineering approach used by Chen et al. [156] should be generally applicable for improving the biotechnological production of ectoines for practical purposes, both in natural and in synthetic cell factories and might, as evidenced by EctB, yield interesting variants of the ectoine biosynthetic enzymes. 

#### 5.2.2. l-2,4-Diaminobutyrate Acetyltransferase EctA

The transformation of DABA and the co-substrate acetyl-coenzyme A into *N*-γ-acetyl-2,4-diaminobutyrate (*N*-γ-ADABA) and CoA is catalyzed by the l-2,4-diaminobutyrate acetyltransferase EctA. This enzyme belongs to the large superfamily of GCN5-related-*N*-acetyltransferases (GNAT) that catalyze the transfer of an acetyl-group from acetyl-coenzyme A as donor to a primary amine as acceptor molecule [174]. Ono et al. [165] were the first to report on the enzymatic properties of an EctA ortholog isolated from *H. elongata*. The partially purified enzyme showed its highest activities at pH 8.2, at temperatures of about 20 °C, and in the presence of 0.4 M NaCl. Gel filtration experiments revealed a native molecular mass of about 45 kDa, which represents a homodimer of the EctA subunit. Three further EctA orthologs from methanotrophic or methylotrophic bacteria (*M. alcaliphilum*, *Methylophaga thalassica*, and *Methylophaga alcalica*) were subsequently biochemically characterized by Trotsenko and co-workers [99,168,175]. Their properties reflect the different physiologies of the host species from which they were isolated. The highest enzyme activities were recorded at a slightly alkaline pH of 8.5 for the enzyme derived from the neutrophilic *M. thalassica* and at a more alkaline pH of 9.5 for the enzymes obtained from the alcaliphiles *M. alcalica* and *M. alcaliphilum*. Interestingly, the activities of the EctA enzymes from the two methylotrophic *Methylophaga* species were inhibited by addition of NaCl or KCl, while the orthologous protein of the methanotrophic *M. alcaliphilum* was activated by these salts with an optimum of salt concentration of about 0.2 M NaCl or 0.25 M KCl [99]. 

**Figure 3:**
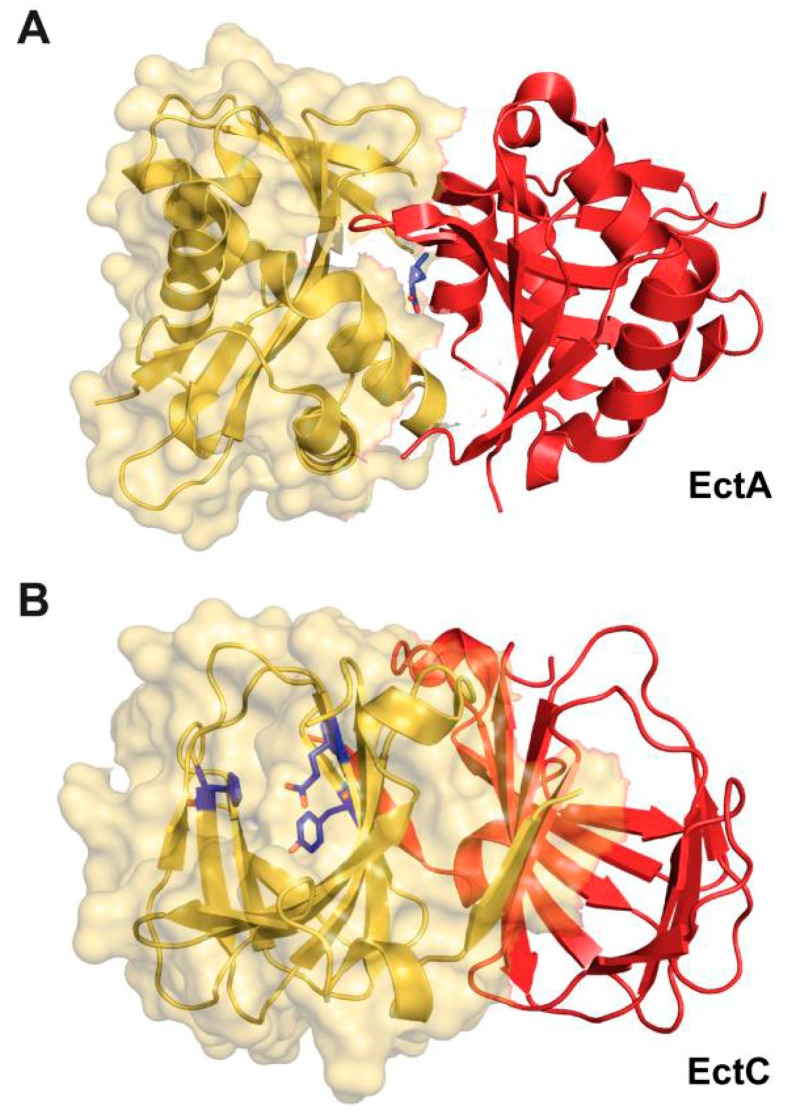

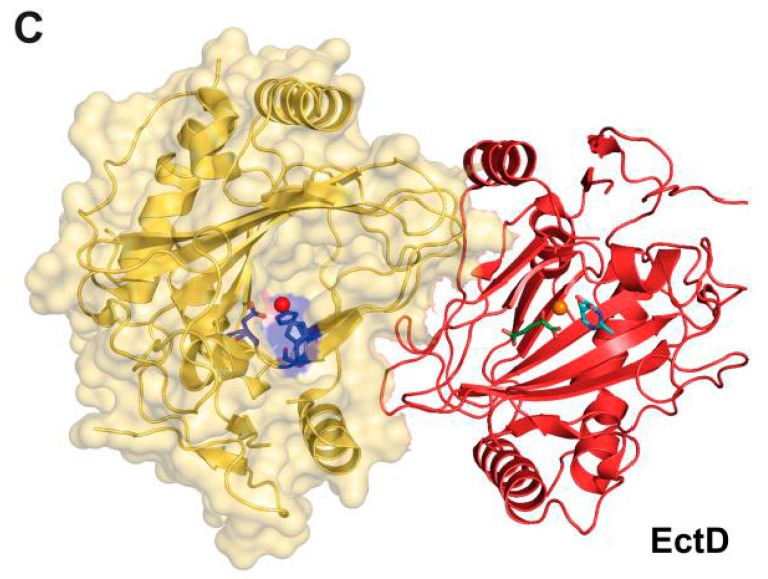
Crystal structures of the EctA and EctC ectoine biosynthetic enzymes and that of the ectoine hydroxylase EctD. Dimers of the l-2,4-diaminobutyrate acetyltransferase (EctA), ectoine synthase (EctC), and ectoine hydroxylase (EctD) are depicted. (**A**) In the crystal structure of the EctA protein from *Bordetella parapertussis* [Protein Data Bank (PDB) accession code 3D3S] a single molecule of the substrate DABA is bound at the dimer interface. (**B**) Crystal structure of the EctC protein from *Sphingopyxis alaskensis* (PDB accession code 5BXX). In one of the dimers, the putative metal-binding residues (Glu^57^, Tyr^85^, His^93^) are highlighted; these protrude into the lumen of the cupin barrel, where the predicted active site of the enzyme is located [176]. (**C**) Crystal structure of the EctD protein from *S. alaskensis* (PDB accession code 4Q5O). In the left monomer of the dimer assembly, the three residues (His^144^, Asp^146^, His^245^) coordinating the catalytically important iron (shown as an orange sphere) are highlighted. In the right monomer of the dimer assembly, the position of the co-substrate for the EctD enzyme, 2-oxoglutarate, and the ectoine-derived product 5-hydroxyectoine are depicted relative to that of the ion catalyst [177].

A crystal structure of the homo-dimeric EctA protein from the human pathogen *Bordetella parapertussis* has been solved [Protein Data Bank (PDB) accession code 3D3S]. In this structure, a single molecule of the substrate DABA is bound within the dimer interface (Figure 3A). However, the experimental details of this particular EctA crystal structure or the biochemistry of the enzyme have not been formally published. Hence, nothing is known about the enzymatic properties of the *B. parapertussis* EctA enzyme and whether the unusual position of the substrate within the dimer assembly was experimentally verified through site-directed mutagenesis of residues within the supposed active site. 

#### 5.2.3. Ectoine Synthase EctC

The last step in ectoine biosynthesis, the ring closure to form the end product ectoine, consists of an intramolecular condensation reaction catalyzed by the ectoine synthase EctC (EC 4.2.1.108) (Figure 2). As a member of the carbon-oxygen hydro-lyases (EC 4.2.1), EctC catalyzes the ring enclosure of ectoine by the elimination of a water molecule from a carbonyl C=O-bond in the substrate *N-*γ-ADABA and the generation of an intramolecular imino bond. 

The ectoine synthase of *H. elongata* [165] shows its highest enzymatic activity at a pH of 8.5–9.0, a temperature of 15 °C, and in the presence of 0.5 M NaCl. The purified enzyme appears to be stabilized in vitro by the presence of high NaCl concentrations since the optimal temperature for the enzyme reaction can be shifted from 15 °C to 30 °C by raising the NaCl concentration from 0.77 M to 3 M. The NaCl concentration also affects the kinetic properties of EctC. The *K*_m_ value of the EctC enzyme for its substrate *N-*γ-ADABA is about 11 mM under low salt concentration (0.05 M NaCl), but decreases to 8.4 mM upon addition of 0.77 M NaCl. The studied EctC enzyme from *H. elongata* showed high substrate specificity towards *N-*γ-ADABA, and Ono et al. [165] found no evidence for a reverse hydrolyzing activity of EctC that would convert the cyclic ectoine molecule into the linear *N-*γ-ADABA. *N-*γ-ADABA (Figure 2) can provide osmostress protection to a degree similar to that afforded by ectoine when it is accumulated in an ectoine synthase (EctC)-deficient mutant of *C. salexigens* [120], or when it is externally provided to salt-stressed *Salmonella typhimurium* cells [178]. Since *N-*γ-ADABA can also protect thermolabile proteins from denaturation [179], it possesses properties that are hallmarks of compatible solutes [54,61,62]. It remains to be seen, however, if this intermediate in ectoine biosynthesis is accumulated in ectoine-producing wild-type strains to cellular levels that would be relevant for notable function-preserving effects. 

The biochemically and structurally best-characterized ectoine synthase is that of the cold-adapted marine alphaproteobacterium *Sphingopyxis alaskensis* [177]. Like other EctC orthologs, it is a dimer in solution, and also in the crystal structures. It possesses the following kinetic parameters: a *K*_m_ of about 5 mM, V_max_ of about 25 U mg^−1^, a *k*_cat_ of about 7 s^−1^. Reflecting the permanently cold habitat of *S. alaskensis*, the temperature optimum of its ectoine synthase is 15 °C, and the enzyme has a pH optimum of 8.5. The optimum salt concentration for the enzyme is around 0.25 M of either KCl or NaCl, but the *S. alaskensis* EctC protein is highly salt-tolerant, as substantial enzyme activity is observed when high concentrations of KCl (up to 1 M) or NaCl (up to 0.5 M) are present in the assay buffer [176]. 

The biochemical properties of the ectoine synthase from the acidiphilic alphaproteobacterium *Acidiphilum cryptum* have been studied, as well [180]. Interestingly, the best enzymatic activity of the recombinantly produced EctC protein was observed in the absence of salt. This difference in the enzymatic properties of the *A. cryptum* ectoine synthase with reference to the strong salt-dependence of the *H. elongata* enzyme (pI 4.87) [165] prompted Moritz et al. [180] to calculate the theoretical isoelectric point (pI) of 80 EctC-type proteins. In this dataset, the *A. cryptum* enzyme exhibits one of the least acidic calculated pI’s (6.03), a feature that might contribute to the salt-independence of this particular ectoine synthase. 

Only a few members of the Archaea are capable of ectoine synthesis (see Section 6.3). One of them is the thaumarchaeon *Nitrosopumilus maritimus* SCM1 [92]. Its ectoine synthase was heterologously produced and biochemically characterized. The enzyme is a dimer in solution and possesses the following kinetic parameters for its natural substrate *N*-γ-ADABA: a *K*_m_ of about 7 mM, a V_max_ of about 13 U mg^−1^, a *k*_cat_ of about 6 s^−1^, and a *k*_cat_/*K*_m_ of about 1 s^−1^ mM^−1^. Its temperature and pH optima are about 30 °C and 7, respectively [92]. While the kinetic parameters of the archaeal EctC enzyme resemble those of its bacterial counterpart from *H. elongata* [165], their enzyme activity profile in response to salt is strikingly different. As outlined above, the *H. elongata* enzyme is strongly dependent on high salinity, while the activity of the *N. maritimus* SCM1 ectoine synthase is restricted to a narrow range of salt concentrations [92]. 

The ectoine synthase can also be exploited to produce non-natural compatible solutes. Witt et al. [181] demonstrated that l-glutamine can be used as an alternative substrate to DABA by the *H*. *elongata* EctC enzyme, albeit with a very low catalytic efficiency. In this reaction, l-glutamine is converted into the cyclic condensation product 5-amino-3,4-dihydro-2H-pyrrole-2-carboxylate (ADPC). ADPC is a synthetic compatible solute as it enhances bacterial growth under salt stress conditions and also stabilizes enzymes against denaturation caused by repeated cycles of freezing and thawing [181]. The EctC-catalyzed formation of ADPC is reversible, with the equilibrium of this reaction lying largely on the site of the hydrolytic product l-glutamine. The *H*. *elongata* EctC enzyme is also able to hydrolyze the synthetic ectoine analogs [143] homoectoine [(*S*)-4,5,6,7-tetrahydro-2-methyl-1H-(1,3)-diazepine-4-carboxylic acid], and DL-DHMICA [(*RS*)-4,5-dihydro-2-methyl-imidazole-4-carboxylic acid], whereas its hydrolytic activity for ectoine was found to be negligible [181]. 

The ectoine synthase belongs to the functionally diverse superfamily of cupin proteins [182,183], and it contains a characteristic cupin domain comprising two conserved motifs [176]. Most members of this protein superfamily are metal-dependent enzymes, and highly conserved residues that are derived from both conserved cupin motifs usually anchor and position the metal cofactor in the active site [182]. Like other cupins [182,183], studies with the *S. alaskensis* EctC enzyme revealed that it is promiscuous with respect to the divalent metal used in enzyme catalysis, but Fe^2+^ is the most-likely biochemically relevant cofactor for the EctC-catalyzed enzyme reaction [176]. 

The crystal structure of the *S. alaskensis* EctC protein (Figure 3B) has been elucidated at a resolution of 1.2 Å (PDB accession codes 5BXX and 5BY5) [176] and exhibits an overall barrel-type fold typical for cupins [182,183]. While the crystal structures of the *S. alaskensis* ectoine synthase are of high resolution, they unfortunately lack the catalytically important metal, and contain neither the substrate *N*-γ-ADABA nor the reaction product ectoine. Bioinformatics and site-directed mutagenesis identified the most likely residues involved in the binding of the catalytically important metal by the *S. alaskensis* ectoine synthase. The corresponding three residues (Glu^57^, Tyr^85^, His^93^) of the *S. alaskensis* EctC protein are evolutionarily highly conserved among a large group of EctC-type proteins. Their side chains protrude into the lumen of the cupin barrel (Figure 3B) [176], the location at which the cyclo-condensation of the *N*-γ-ADABA substrate to ectoine will take place [182,183]. The *S. alaskensis* EctC protein is a head-to-tail dimer; the dimer interface is formed by two anti-parallel β-sheets present near the N- and C-termini of each monomer, stabilizing interactions that thus occurs twice within the EctC dimer assembly (Figure 3B). 

#### 5.2.4. Ectoine Hydroxylase EctD

A substantial number of the ectoine producers additionally synthesize 5-hydroxyectoine [92,93,98] through a position- and stereo-specific hydroxylation of ectoine (Figure 2). Bursy et al. [79,98] elucidated the biochemical basis for the formation of 5-hydroxyectoine through studies with the purified ectoine hydroxylases (EctD; EC 1.14.11.55) from the moderate halophile *S. salexigens* (taxonomically now reclassified as *Virgibacillus salexigens*) and the soil bacterium *S. coelicolor*. This biochemical analysis and subsequent structural work [177,184] revealed that EctD is a member of the superfamily of non-heme Fe(II)-containing and 2-oxoglutarate-dependent dioxygenases [185]. The O_2_-dependent hydroxylation of the substrate ectoine is accompanied by the oxidative decarboxylation of 2-oxoglutarate to form succinate and CO_2_, while the iron cofactor acts as a catalyst for the activation of molecular oxygen [186] (Figure 2). Therefore, the catalytic activity of the EctD enzyme is strongly dependent on the presence of molecular oxygen [92,93,177,184]. 5-hydroxyectoine produced in vivo by *S. parvulus* is known to have the (4*S*,5*S*) stereo-chemical configuration [90], and the very same configuration is also found in the reaction product formed in vitro by the purified *V. salexigens* EctD enzyme as analyzed by one-dimensional ^1^H-NMR spectroscopy [98]. 

To date, nine ectoine hydroxylases have been biochemically characterized; eight of these originate from various, mostly extremophilic, bacteria (*V. salexigens*, *S. coelicolor*, *S. alaskensis*, *Paenibacillus lautus*, *P. stutzeri*, *Alkalilimnicola ehrlichii*, *A. cryptum*, *H. elongata*) [93,184], and one of the studied enzymes was derived from the archaeon *N. maritimus* SCM1 [92]. The EctD-containing microorganisms live in ecophysiologically rather different habitats, but the biochemical properties of the studied ectoine hydroxylases are all very similar. Their enzyme activities are not strongly dependent on salts, and their pH (between 7.5 and 8) and temperature optima (between 32 and 40 °C) range within narrow windows. The apparent kinetic parameters of these enzymes for the substrate ectoine (*K*_m_ values between 6 and 10 mM) and the co-substrate 2-oxoglutarate (*K*_m_ values between 3 and 5 mM) are similar, and their catalytic efficiencies (*k*_cat_/*K*_m_) vary only between 0.12 and 1.5 mM^−1^ s^−1^ [92,93,184]. Hence, ectoine hydroxylases possess rather moderate affinities for their substrate ectoine, a property that is potentially connected with the fact that the accumulation of ectoine to a substantial intracellular level via de novo synthesis typically precedes the production of 5-hydroxyectoine [79,98,122,123]. Although all ectoine hydroxylases studied to date possess similar kinetic parameters, it should be noted that the in vivo performance of these enzymes can differ substantially when they are expressed in an *E. coli*-based synthetic cell factory that imports externally provided ectoine via the osmotically induced ProP and ProU osmolyte import systems [187,188,189], hydroxylates it, and then excretes the newly formed 5-hydroxyectoine almost quantitatively into the growth medium [151]. These differences in performance might stem from differences in the production levels or the stability of the recombinant proteins in the heterologous host, or the properties of the *E. coli* cytoplasm is not optimal for the enzymatic activities of the various EctD proteins. Such differences in the in vivo performance of ectoine hydroxylases with seemingly similar in vitro kinetic parameters need to be carefully considered when such enzymes are used in heterologous microbial cell factories for the biotechnological production of 5-hydroxyectoine [116]. 

Among the four enzymes involved in ectoine/5-hydroxyectoine biosynthesis [164,165,166], the ectoine hydroxylase is certainly the best studied [92,93,177,184,186]. A substantial number of EctD enzymes have been biochemically assessed that were derived from physiologically and taxonomically distinct groups of microorganisms [92,93]. Furthermore, the structure/function relationship of this enzyme has been studied by site-directed mutagenesis, by molecular dynamics simulations and finally via crystal structure analysis [93,177,184,186]. Together, these studies have led to a detailed understanding of the EctD-mediated enzyme reaction [186] and illuminated the architecture of the active site [177]. Crystal structures of *V. salexigens* without any substrates or products [184], and that of *S. alaskensis* with various ligands [177] have been reported. 

The ectoine hydroxylase is a dimer in solution and in the crystal structure. The dimer interface of the swapped head-to-tail dimeric structure is primarily formed through interactions by loop areas pointing from one monomer towards the other (Figure 3C). bona fide EctD-type proteins can be distinguished from other members of the broadly distributed non-heme Fe(II)-containing and 2-oxoglutarate-dependent dioxygenases superfamily through an evolutionarily highly conserved signature sequence consisting of a continuous stretch of 17 amino acids (F-x-W-H-S-D-F-E-T-W-H-x-E-D-G-M/L-P) [177,184]. When the signature amino acid sequence is viewed in the context of the EctD crystal structure, this segment of the EctD polypeptide chain is important from a structural point of view, as it forms one side of the cupin barrel (Figure 3C). In addition, it also contains five residues involved in the binding of the iron catalyst, the co-substrate 2-oxoglutarate, and the reaction-product 5-hydroxyectoine [177,184]. 

In their excellent and widely appreciated overview on ectoines as stress protectants and commercially interesting compounds, Pastor et al. [91] suggest that 5-hydroxyectoine may also be formed by first converting the EctA-formed *N*-γ-acetyl-2,4-diaminobutyrate (Figure 2) into 3-hydroxy-*N-*γ-acetyl-2,4-diaminobutyrate, which is proposed to be subsequently cyclized to 5-hydroxyectoine. In this envisioned pathway, the activity of EctC is circumvented by an unknown enzyme and the existence of an additional unknown enzyme is invoked that would cyclize the linear 3-hydroxy-*N-*γ-acetyl-2,4-diaminobutyrate molecule to 5-hydroxyectoine [80]. This proposal for an alternative route for the formation of 5-hydroxyectoine is primarily based on the properties of a particular *ectC* mutant (*ectC*:*Tn*1732; strain CHR63) of *C. salexigens* [120] in which, quite surprisingly, both ectoine and 5-hydroxyectoine were still detected [179]. There have been no follow-up studies on this hypothetical 5-hydroxyectoine biosynthetic route since it was originally proposed by Canovas et al., in 1999 [179]. Synthesis of ectoine and 5-hydroxyectoine in the *ectC* mutant may be a particular feature of the studied *C. salexigens* genetic background [120,179], or the fact that *C. salexigens* is also able to catabolize ectoines [80,190] and may thus use some of the degradative enzymes (see Section 7) to partially restore ectoine/5-hydroxyectoine production. 

We suggest that the envisioned EctC- and EctD-independent route for the synthesis of 5-hydroxyectoine [91] is of no physiological relevance in natural settings of osmotically stressed wild-type 5-hydroxyectoine-producing microorganisms. To avoid confusion, this hypothetical pathway should, in our view, not be presented in the literature [80,91] as a true alternative to the biochemically and structurally buttressed direct and stereo-specific hydroxylation of ectoine by the ectoine hydroxylase EctD [92,93,98,177] until it is further substantiated by molecular and biochemical evidence. 

#### 5.2.5. Specialized Aspartokinase Ask_Ect

The precursor for ectoine synthesis (Figure 2), l-aspartate-β-semialdehyde, is a central metabolic hub in microorganisms from which a branched network of various biosynthetic pathways diverges [167]. l-aspartate-β-semialdehyde is synthesized through the sequential enzymatic reactions of an aspartokinase (Ask; EC 2.7.2.4) and a l-aspartate-semialdehyde-dehydrogenase (Asd; EC 1.2.1.11). Ask synthesizes l-4-aspartyl-β-phosphate via an ATP-dependent phosphorylation of l-aspartate, which is then in turn reduced to l-aspartate-β-semialdehyde by the Asd enzyme in an NADPH-dependent reaction (Figure 2). To avoid a wasteful production of the energy-rich intermediate l-4-aspartyl-β-phosphate, the enzymatic activities of aspartokinases are usually regulated by feedback inhibition and the expression of the corresponding *ask* gene is also often subjected to sophisticated transcriptional regulation [167]. Since major production routes of biotechnologically interesting antibiotics and commercially used amino acids (e.g., l-lysine) branch off from l-aspartate-β-semialdehyde as the initial metabolite, aspartokinases are often targeted in genetic engineering approaches to relieve their feedback inhibition. This leads to an increased cellular l-aspartate-β-semialdehyde pool and thereby fosters the flow of this precursor into biosynthetic pathways of interest [152,191]. When applied to the heterologous production of ectoine in *E. coli,* a bacterium that does not naturally synthesize ectoine [187], the yield was indeed improved by co-expressing a feedback-resistant aspartokinase (LysC) derived from *Corynebacterium glutamicum* together with the ectoine biosynthetic genes obtained from *Marinococcus halophilus* [191]. Such feedback-resistant aspartokinases have also been employed in the design of engineered synthetic microbial cell factories, thereby resulting in enhanced production of ectoines [152,153,155]. 

Because the feedback-control of Ask enzyme activity could potentially lead to a bottleneck in ectoine biosynthesis [191], the report of Reshetnikow et al. [168] that the osmotically inducible ectoine biosynthetic gene cluster of *M. alcaliphilum* was co-transcribed with a gene encoding an aspartokinase was of considerable interest. This finding indicated that the enzyme encoded by this particular *ask* gene could play a specialized role in ectoine biosynthesis. Indeed, it was observed in subsequent studies [93] that a considerable number of ectoine/5-hydroxyectoine biosynthetic gene clusters include an additional paralogous *ask* gene (referred to in the following as *ask_ect*) [122] (see Section 5.3).

A comprehensive cohesion group analysis of aspartokinases revealed that the Ask_Ect enzymes form a distinct sub-cluster among the large aspartokinase enzyme family, and that those residues implicated in participating in the feedback control of various Ask enzymes are not conserved in the Ask_Ect group [167]. Stöveken et al. [122] purified such an Ask_Ect enzyme from the ectoine/5-hydroxyectoine-producing plant-root-associated bacterium *P. stutzeri* A1501 and benchmarked its biochemical properties against those of the biosynthetic standard aspartokinase (Ask_LysC) present in this bacterium as well. Both enzymes possess similar kinetic parameters, but exhibit significant differences with regard to the allosteric control by biosynthetic products derived from l-aspartate. Ask_LysC was inhibited by l-threonine alone and in a concerted fashion by l-threonine and l-lysine, whereas Ask_Ect showed inhibition only by l-threonine. Moreover, the inhibiting effect by l-threonine on the latter enzyme was significantly reduced when the enzyme activity assay was carried out in presence of 650 mM NaCl or KCl [122]. 

**Figure 4:**
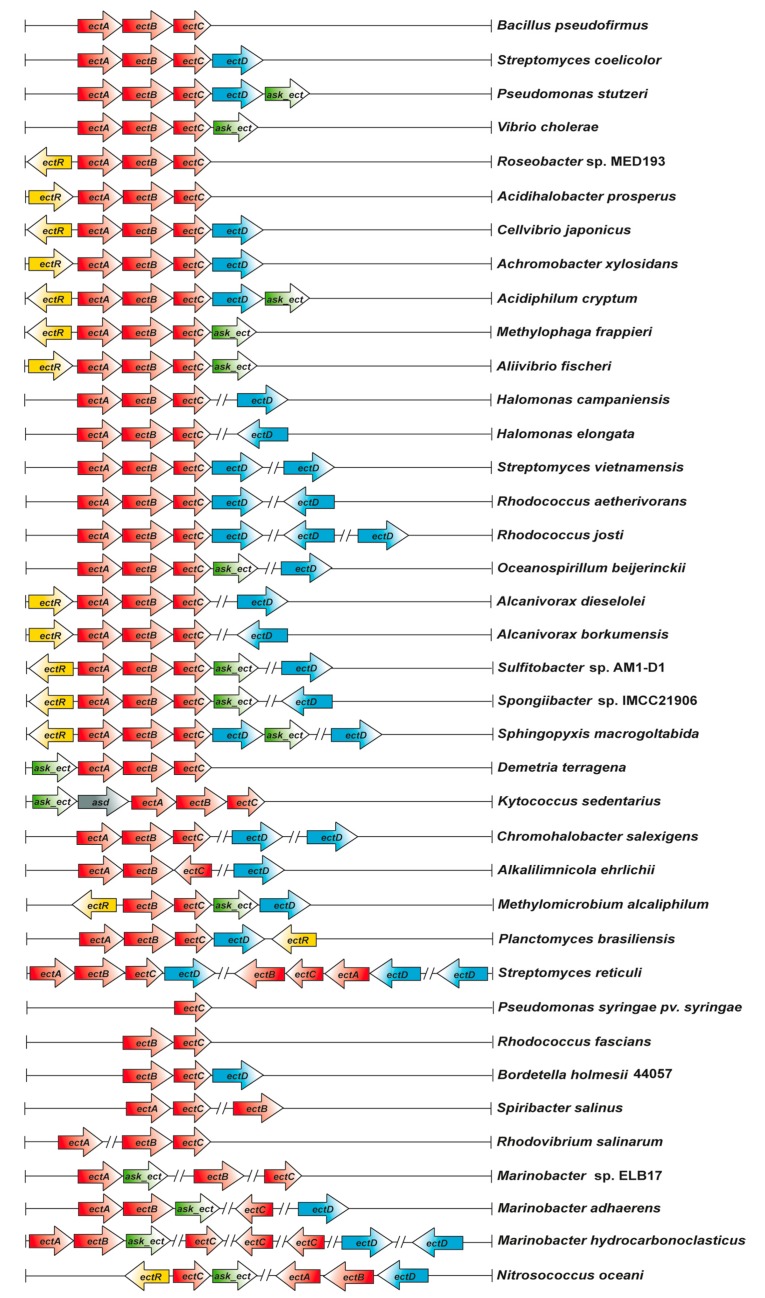
Diversity of the genetic organization of ectoine and 5-hydroxyectoine biosynthetic gene clusters in microbial genomes.

**Figure 5:**
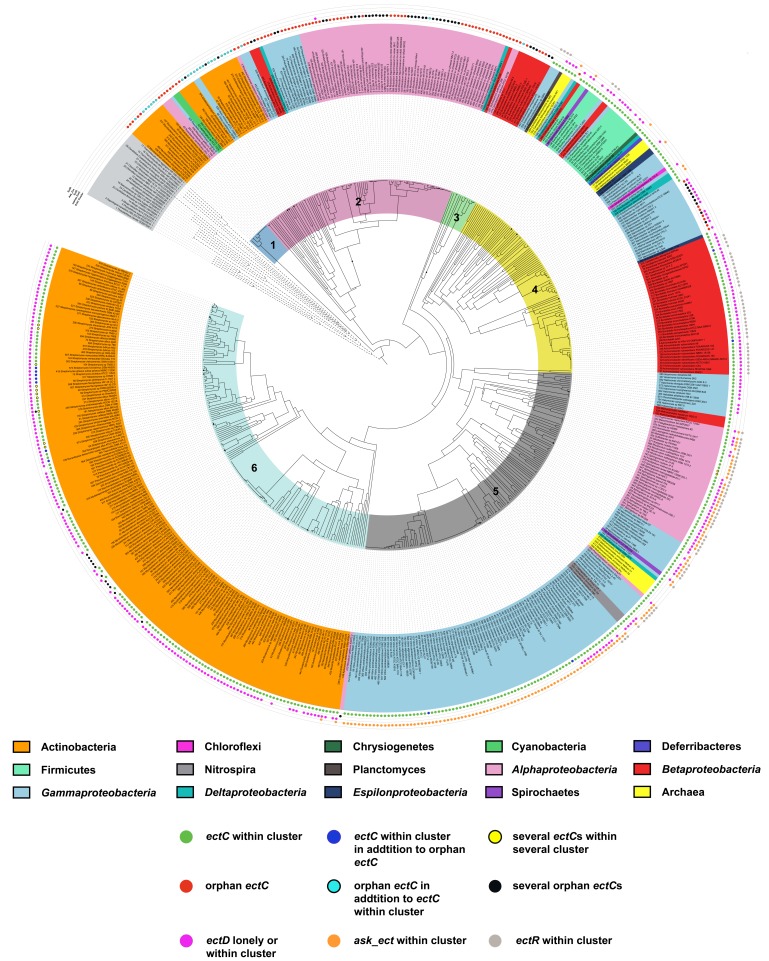
Phylogenomics of the ectoine synthase. The amino acid sequences of 582 EctC-type proteins were retrieved from microorganisms with fully sequenced genomes, aligned with MAFFT [192] and then used for a clade analysis using the iTOL software [193]. The tree was rooted with a number of microbial cupin-type proteins, a superfamily of proteins [182,183] to which the EctC protein also belongs [176]. The phylogenetic affiliation of the various EctC proteins is depicted in different colors shown in the outer ring, and the color code is explained in the figure. Different groups (1 to 6) in which the EctC-type proteins can be clustered are depicted in the inner colored circle. The dots in the outmost 5 rings depict (from the inside to the outside) if the EctC protein is encoded within an *ect* biosynthetic gene cluster, if the EctC protein is an orphan, if the pertinent EctC-containing microorganism also possesses the ectoine hydroxylase EctD, if the specialized aspartokinases Ask_Ect is part of the *ect* cluster, or if the *ect* gene cluster is affiliated with a gene encoding the EctR regulatory protein.

An *E. coli* strain carrying the plasmid-based *ectABCD-ask_ect* gene cluster from *P. stutzeri* A1501 produced substantially more (about 5-fold) ectoine/5-hydroxyectoine than a strain expressing the same gene cluster without the *ask_ect* gene [122]. Taken together, these findings suggest that the *ask_ect* gene encodes an aspartokinase with a specialized role for the biosynthesis of ectoine and 5-hydroxyectoine. The frequent co-expression of this gene with osmotically inducible *ect* gene clusters [93,122,168] (Figure 4 and Section 6.3) will ensure an optimal supply of the precursor l-aspartate-β-semialdehyde under osmotic stress conditions. However, it should be noted that the majority of ectoine/5-hydroxyectoine-producing bacteria do not contain such a specialized Ask_Ect enzyme (Figure 5), indicating that they may use different strategies to maintain their l-aspartate-β-semialdehyde pools at high enough cellular levels to support their large-scale ectoine/5-hydroxyectoine biosynthetic activities under high-salinity growth conditions. 

#### 5.2.6. Adjusting Central Carbon Metabolism to the Drain Exerted by Ectoine Biosynthesis

Under osmotic stress conditions, ectoines can be accumulated through synthesis to exceedingly high intracellular concentrations [91,94], and the degree of the imposed osmotic stress dictates their pool size. There seems to be a linear relationship between the external osmolarity/salinity and the amounts of the produced ectoines [116,117,118]. As a consequence, the microbial cell has to sensitively adjust its metabolism to constraints imposed by high-level synthesis of the nitrogen-rich ectoine/5-hydroxyectoine molecules (Figure 1B), which will impose a serious drain of available carbon- and nitrogen-sources. Hence, it is necessary to understand the interplay of the carbon and nitrogen supplies for the production of ectoines in greater detail [194]. Their synthesis burdens the assimilation of nitrogen via the glutamine synthetase pathway and central metabolic routes by recruiting TCA-cycle intermediates—in particular, oxaloacetate and acetyl-CoA [195,196,197]. Consequently, anaplerotic routes have to be engaged to replenish the TCA cycle for routine central carbon metabolism and at the same time an increased flux of metabolites into the ectoine/5-hydroxyectoine biosynthetic pathway has to be ensured. Genome-scale modeling and integrative systems biology approaches have recently provided insights into how this is accomplished by *C. salexigens* [196] and *H. elongata* [197]. These studies paint a complex picture of the involved metabolic changes and highlight the considerable metabolic and energetic burden [24,37,106] that osmotically stressed cells face when they try to alleviate osmotically imposed constraints on growth through the synthesis of stress-relieving ectoines [194,195,196,197]. This aspect is not only important for a full understanding of the cells’ behavior under osmotic stress conditions, but is also a pre-requisite to further improvement of the high-yield production of ectoines by natural and synthetic microbial cell factories. 

## 6. Genetics and Phylogenomics of Ectoine and 5-Hydroxyectoine Biosynthetic Genes

### 6.1. Genetic Organization of the Ectoine/5-Hydroxyectoine Biosynthetic Gene Clusters

The description of the *ectABC* genes in *M. halophilus* [95], along with that of the *ectABCD* locus in *Streptomyces chrysomallus* [96], provided the primers for a molecular analysis of the ectoine/5-hydroxyectoine biosynthetic genes. Studies on *C. salexigens* [97] and *S. salexigens* [98] subsequently demonstrated that the *ectD* gene was not necessarily part of the *ectABC* gene cluster but could be encoded somewhere else in the genome, with *C. salexigens* possessing even two *ectD*-type genes [80,97] (Figure 4). Previous genome assessments [91,92,93,99], and our current own comprehensive database searches (see Section 6.3), revealed an evolutionarily rather conserved genetic configuration of the ectoine/5-hydroxyectoine biosynthetic genes in many bacterial and some archaeal genomes. In some notable cases, several copies of complete ectoine/5-hydroxyectoine biosynthetic gene clusters are even present that might have arisen either through gene duplication or lateral gene transfer. *Streptomyces reticuli* is an example where two copies of the *ectABCD* gene clusters are present, and an additional copy of an *ectD*-type can even be found in the genome of this actinobacterium. The occurrence of multiple copies of *ectD*-type genes in the same genome is not unusual (Figure 4). 

As highlighted in Figure 4, the *ectABC* and *ectABCD* gene clusters build a conserved backbone in most ectoine/5-hydroxyectoine producers that can additionally be genetically configured with the gene (*ask_ect*) for the specialized aspartokinases and/or the gene (*ectR*) for a MarR-type regulator, EctR (see Section 5.2) [91,92,93,99,122,168,198]. In practically all ectoine/5-hydroxyectoine gene clusters inspected by us, we found that the gene for the second enzyme (l-aspartate-β-semialdehye-dehydrogenase, Asd) involved in providing the ectoine biosynthetic precursor l-aspartate-β-semialdehyde is absent (Figure 2 and Figure 4). The notable exception to this rule is the *ect* gene cluster from the marine actinobacterium and opportunistic pathogen *Kytococcus sedentarius* where *ask_ect* and *asd* are encoded up-stream of the *ectABC* operon (Figure 4).

In addition to the evolutionarily conserved *ectABC/ectD* gene arrangement, substantially re-arranged configurations of the *ect* genes can be found in a sizable number of microorganisms (Figure 4). There can be a re-arrangement of individual genes within the *ect* cluster, but there are also cases where individual *ect* genes have been separated from each other, or where multiple copies of the same gene (e.g., *ectC*) are present at various locations within the genome (Figure 4).

Many representatives with re-arranged or disentangled *ect* biosynthetic genes live in marine ecosystems [199]. Given the re-arrangement of the canonical *ect* gene configuration in these bacteria, one wonders if they are capable of ectoine/5-hydroxyectoine production. One representative of this group of microorganisms is the gammaproteobacterium *Spiribacter salinus*, an ecophysiologically successful and abundant inhabitant of hypersaline ecosystems [200]. In its genome, *ectAB* and a separate *ectC* gene can be found (Figure 4); despite this non-canonical arrangement of the *ect* biosynthetic genes, a recent study demonstrated the production of ectoine in *S. salinus* in response to increases in the external salinity [199]. 

### 6.2. Regulation of ect Gene Expression

It is fitting from the main physiological function of ectoines as osmostress protectants that the transcription of the corresponding biosynthetic genes is under osmotic control. Indeed, studies with reporter gene fusions and Northern-blot analysis have demonstrated that this is the case in both Gram-negative and Gram-positive bacteria. However, the way osmotic stress is sensed by the bacterial cell and the way the gleaned information is processed to trigger enhanced *ect* transcription is far from understood. As a matter of fact, the literature pertinent to this topic is plagued with a considerable over-interpretation of preliminary findings.

Northern-blot analysis of osmotically stressed *V*. (*Salibacillus*) *salexigens* cells proved that transcription of the *ectABC* genes, and of the separately encoded *ectD* gene, is strongly enhanced in high-salinity growth media. Primer extension analysis pinpointed a single *ectABC* promoter that resembles in its sequence typical SigA-type promoters [98], the housekeeping sigma factor of Bacilli [201]. Transcription of the *ectABC* genes from *V. pantothenticus* was found to be responsive both to increases in osmolarity and to decreases (but not to increases) in growth temperature [77]. In this Gram-positive bacterium, transcription of the gene for the ectoine/5-hydroxyectoine transporter EctT followed the same pattern of gene expression, and primer extension analysis demonstrated that this response is mediated by a single SigB-type promoter [78]; SigB is the general stress-responsive alternative sigma factor in Bacilli [202] and salt and temperature stress are major inducers of the SigB-regulon in *B. subtilis* [203]. The dependence of the *V. pantothenticus ectT* gene on SigB was verified in a *sigB* mutant of *B. subtilis* [78], but the implicated dependence of *ectABC* transcription on SigB activity in *V. pantothenticus* was not experimentally tested [77]. 

When the DNA sequence of the first ever cloned *ectABC* gene cluster was reported by Louis and Galinski [95], these authors proposed that its expression was mediated by a single SigB-dependent promoter positioned upstream of *ectA*. Because *M. halophilus* is a Gram-positive bacterium, this was a reasonable assumption, but no experimental evidence for the involvement of SigB was provided in this study [95]. However, in view of the fact that *E. coli* does not possess SigB and that SigB-dependent promoters differ substantially from the consensus sequence of promoters recognized by the house keeping sigma factor RpoD or the general stress alternative sigma factor RpoS of *E. coli* [204,205], it was rather surprising that the introduction of the *M. halophilus* recombinant *ect* genes into this Gram-negative host bacterium led to an osmostress-responsive production of ectoine [95]. Bestvater and Galinski [206] subsequently rationalized this finding by invoking the fortuitous existence of stationary-phase/general stress-type RpoS-dependent promoters in front of the *M. halophilus ectABC* genes. If these types of promoters exist, they certainly cannot have any physiological relevance in the authentic *M. halophilus* host because Gram-positive bacteria do not possess RpoS-type alternative sigma factors [205]. Hence, the dependence of the *M. halophilus ectABC* gene cluster on SigB awaits experimental verification.

In *H. elongata*, the industrially used bacterium for the production of ectoines [86,91,94], two promoters preceding the *ectABC* genes and an additional promoter present in front of *ectC* were mapped by RACE-PCR [158]. Based upon DNA-sequence inspection, Schwibbert et al. [158] suggested that the promoter exclusively driving *ectC* transcription was recognized by the alternative transcription factor Sig-54, a sigma factor that is frequently involved in regulating the expression of genes involved in physiological processes connected to nitrogen metabolism. However, it is not obvious to us what the function of this internal promoter within the *H. elongata ect* gene cluster might be; its implicated dependence on Sig-54 activity was not verified experimentally [158]. One of the two promoters present in front of *ectA* was described by Schwibbert et al. [158] as a promoter recognized by the housekeeping sigma factor RpoD (Sig-70); the distal located promoter was deemed to be dependent or the stationary-phase/general stress sigma factor RpoS (Sig-38). While the putative RpoS-dependent promoter of the *H. elongata ect* gene cluster exhibited features found in some other osmotically regulated RpoS-dependent promoters from *E. coli* [207,208], the *H. elongata* promoter nevertheless deviates considerably (in particular in the spacing of the -10 and -35 regions) from typical RpoS-type promoters [130,131,205]. Osmoregulation of either the proposed RpoD- or RpoS-dependent *H. elongata ect* promoters was not studied in any detail, nor was the involvement of RpoS in *ect* gene expression verified by mutant analysis [158]. 

The importance of a careful genetic analysis is exemplified by data reported on the apparent complex transcriptional control of the *ectABC* genes from *C. salexigens*, a salt-tolerant bacterium closely related to *H. elongata* [80,118]. S1 mRNA protection assays suggested the existence of four promoters driving *ectABC* transcription, three of which were deemed by Calderon et al. [118] to be osmotically responsive. When a *ectA-lacZ* reporter fusion expressed from the three promoters mapped in front of *ectA* was introduced into *E. coli*, expression of the reporter fusion was linearly dependent on the osmotic strength of the growth medium and its activity increased strongly in stationary phase. One (P*ectA*-3) of the suggested promoters of the *C. salexigens* biosynthetic *ect* gene cluster [118] resembled, with respect to certain features of the -10 and -35 regions, osmoregulated *E. coli* promoters that are dependent on the alternative sigma-factor RpoS [130,131,205,207,208]. Since the activity of above described *ectA-lacZ* reporter fusion carrying all three promoters was reduced by about 50% in an *E. coli rpoS* mutant, Calderon et al. [118] ascribed an important role to this alternative sigma-factor for the direct transcriptional regulation of the *C. salexigens ect* gene cluster in the heterologous *E. coli* host. However, subsequent follow-up studies by the same laboratory showed that the observed effect of RpoS was indirect [209]; in other words, the initially envisioned direct effect of RpoS on the proposed *C. salexigens* P*ectA*-3 promoters does not exist. 

In studying the interplay between iron homeostasis and the salt stress response of *C. salexigens*, Argandona et al. [135] found that the amount and relative proportion of ectoine and 5-hydroxyectoine was affected by excess iron in the growth medium. These authors ascribed an activator function of the Fur regulatory protein for the transcription of the ectoine biosynthetic genes, and through in silico inspection of the *ect* regulatory region, noted the presence of several potential Fur DNA-binding boxes overlapping two of the putative *ect* promoters. In quantitative RT-PCR experiments, they observed a drastic fall in the *ectA* transcript in a *fur* mutant [135], consistent with previous *ectA-lacZ* transcriptional reporter fusion studies that revealed a down-regulation of *ect* expression in *C. salexigens* wild-type cells grown at high salinity in the presence of excess iron [118]. However, the data reported by Argandona et al. [135] on the suggested direct interaction of the Fur protein with the *ect* regulatory region and the proposed activator function of the Fur regulatory protein are hard to reconcile with the findings of these authors that there was no real difference in ectoine/hydroxyectoine content between the *C. salexigens* wild-type and its isogenic *fur* mutant [135]. Hence, the inferred interaction of Fur with the *ect* promoter region and the role of Fur as an activator of *ect* transcription [135] awaits verification through DNA-binding studies and mutational analysis.

The S1 mRNA protection data reported by Calderon et al. [118] also suggested the existence of a heat-shock (RpoH; Sig-32)-dependent promoter (PectB) that is positioned upstream of the *C. salexigens ectB* gene. The physiological rationale for producing a separate *ectB-ectC* transcript under heat-shock conditions is not immediately apparent but reporter gene fusion studies showed enhanced expression of a *PectB-lacZ* reporter fusion at high temperature (40 °C) [118]. However, a molecular analysis that would identify this promoter as a direct target for RNA-polymerase complexed with the alternative sigma factor RpoH was not performed. 

We generally consider the assignment of putative *ect* promoters that are in their core exclusively based on DNA-sequence gazing as unreliable, and we caution against the over-interpretation of such suggestions in published reports. In our view, reliable data on the transcriptional regulation of *ect* genes can only be attained through site-directed mutagenesis of the proposed promoter(s) and, if an alternative sigma factor is invoked in their transcriptional activity, through studies with appropriate mutant strains (if at all possible) in the authentic ectoine/5-hydroxyectoine producer bacterium. 

DNA sequence inspection can readily overlook the true osmotically controlled promoter(s) of *ect* biosynthetic genes, as these might deviate considerably from the consensus sequences one might look for. In a recent report, Czech et al. [116] studied the osmostress-responsive transcription of the *ect* biosynthetic genes from the plant-root-associated Gram-negative bacterium *P. stutzeri* A1501 in heterologous *E. coli* host strains. While the *ect* promoter possesses a good match (TTGAGA) to the consensus sequence (TTGACA) of the -35 element of Sig-70-type *E. coli* promoters [204], its highly G/C-rich -10 sequence (TACCCT) [116] deviates strikingly from the A/T-rich consensus sequence (TATAAT) of these types of promoters. Furthermore, the spacing of the -10 and -35 elements of the *ect* promoter with a length of 18 bp was sub-optimal for Sig-70-type *E. coli* promoters [204]. Osmostress-responsive promoters with such G/C-rich -10 elements and sub-optimal spacer length have previously been described both in *E. coli* and *B. subtilis* [84], but no *ect* promoter has been reported with such an unusual configuration in its -10 region. This prompted the study of the salient features of this promoter through *lacZ* reporter gene studies and extensive site-directed mutagenesis experiments [116]. The transcriptional activity of a wild-type *ect-lacZ* reporter fusion, when introduced into *E. coli*, proved to be linearly dependent on the external salinity and responded to true osmotic cues, as both ionic (NaCl, KCl) and non-ionic (suchrose, lactose) osmolytes triggered similar increases in promoter activity [116]. Osmotic induction of the *ect-lacZ* reporter fusion required the establishment of an osmotically active trans-membrane gradient, as high concentration of membrane-permeable glycerol did not trigger enhanced *ect* promoter activity [116]. 

Site-directed mutagenesis studies proved that the *P. stutzeri ect* promoter was critically dependent for its activity on the function of the *E. coli* house-keeping Sig-70 transcription factor. Furthermore, point mutations rendering its -35 and -10 regions, or that of the spacer length, towards a closer match to the consensus sequence, conferred drastic changes in gene expression. Typically, the activity of the mutant *ect* promoters rose substantially under both non-salt and salt-stress conditions [116]. Studies with *E. coli* mutants with defects in *hns*, *rpoS*, *ompR*, or *cya*, genes that have been implicated in osmoregulation of various *E. coli* genes demonstrated that the *P. stutzeri ect* promoter operates in its osmotic control completely independently of these important transcription factors [116].

None of the 18 variants of the *P. stutzeri ect* promoter constructed by site-directed mutagenesis lost osmotic control altogether; surprisingly, this was even true for an *ect* promoter variant that was synthetically adjusted to the complete consensus sequence of Sig-70 *E. coli* promoters [116]. Hence, one can conclude from this study that (i) the deviations of the *P. stutzeri ect* promoter from the consensus sequence serve to keep promoter activity low when the cell does not have to rely on the synthesis of ectoines, while simultaneously allowing strong osmotic induction of *ect* transcription when the cell physiologically needs these cytoprotectants for its adjustment to the adverse environmental conditions; and that (ii) a determinant for osmotic control must be present outside the particular sequence of the -10 and -35 regions and of the spacer that separates them. Osmotic control of the *P. stutzeri ect* promoter was traced through deletion analysis to a 116-bp DNA fragment [116]. Hence, despite the fact that *E. coli* does not synthesize ectoines naturally [187], the *P. stutzeri ect* promoter retained its exquisitely sensitive osmotic control in the heterologous host bacterium, indicating that osmoregulation of this promoter is an inherent feature of the rather small regulatory region per se. It is not yet clear yet how this can be accomplished mechanistically, but Czech et al. [116] speculated that RNA polymerase alone, perhaps in response to changes in osmotically triggered changes in DNA supercoiling [210], and in combination with changes in the intracellular ion pool (in particular the pair of K^+^ and l-glutamate) [207,208,211] and the size of the compatible solute pool [212,213], might afford osmoregulation of *ect* expression. It is currently difficult to grasp intuitively that the exquisitely sensitive osmotic control of the *ect* promoter and the tuning of its strength via incremental increases in sustained osmotic stress can be explained by this molecular mechanism alone. It will be a challenge to experimentally verify or refute this model through in vivo or in vitro studies. 

A highly interesting finding with respect to the genetic control of *ect* genes is the report of Mustakhimov et al. [198], who studied these biosynthetic genes in the halotolerant methanotroph *M. alcaliphilum* 20Z. These authors detected a gene (*ectR*) positioned upstream of the *ectABC-ask_ect-ectD* gene cluster (Figure 4) that encodes a MarR-type regulator, a super-family of widely distributed transcription factors [214]. Primer extension analysis showed that the osmoregulated *ect* genes of *M. alcaliphilum* 20Z are expressed from two closely spaced promoters. Through foot-printing analysis, Mustakhimov et al. [198] found that EctR binds a homodimeric protein to a region overlapping the -10 region of the promoter most distal to the beginning of the *ectA* gene. EctR acts as a repressor of *ect* expression in *M. alcaliphilum* 20Z but notably, salt-stress responsive induction of the *ect* gens still occurred in an *ectR* mutant strain [198]. Hence, EctR is certainly not solely responsible for osmotic induction of the *ect* genes. Interestingly, EctR controls the transcription of its own gene in *M. alcaliphilum* 20Z [198]. 

In the methanol-utilizing bacterium *M. alcalica*, EctR served as a repressor for the ectoine biosynthetic gene cluster as well [215], and in the methylotroph *Methylophaga thalassica*, the purified EctR protein interacted in DNA-band shift assays with a region carrying the two promoters of the *ect* biosynthetic genes. However, in contrast to the situation in *M. alcaliphilum* 20Z, no auto-regulation of *ectR* transcription was found [99]. In both *M. alcaliphilum* 20Z and *M. thalassica*, the level of the *ectR* transcript increased upon osmotic up-shock and a complex array of three intertwined promoters was found to direct the transcription of the *M. thalassica ectR* gene [215]. 

All currently available data point to the function of EctR as a repressor of ectoine biosynthesis genes. Unfortunately, the cellular or environmental cues to which this interesting regulatory protein reacts are not known. It seems possible that EctR responds to changes in the ionic/osmotic strength of the cytoplasm. Such a mechanism has been proposed for the BusR regulatory protein, which regulates the expression of an operon (*busAA-busAB*), encoding an ABC-type compatible solute import system in *Lactococcus lactis* [216,217], and for the CosR regulator controlling (among other genes) genes for ectoine biosynthesis and compatible solute import in *Vibrio cholerae* [218]. Our database searches (see Section 6.3) revealed that *ectR*-type genes are found in close proximity to *ect* biosynthetic genes in 19% (97 out of 510) of putative ectoines producers and that all of the *ectR*-harboring microorganisms belong to members of the *Alphaproteobacteria*, *Betaproteobacteria*, and *Gammaproteobacteria* (Figure 5). Previous phylogenetic analysis of EctR-type proteins conducted by Reshetnikov et al. [99] and Mustakhimov et al. [215] showed that they form a specific phylogenetic subgroup with in the very large superfamily of MarR-type transcriptional regulators [214]. Like other MarR-type regulatory proteins, EctR is predicted to contain a winged-helix-turn-helix DNA-binding motive, and the EctR operator sequence in *M. alcaliphilum* 20Z, as revealed by DNA-foot-printing analysis, comprises a pseudo-palindromic highly A/T-rich DNA-sequence composed of two eight-bp half-sites separated by two bp [198,215]. 

In the context of discussions on the genetic control of the ectoine/5-hydroxyectoine biosynthetic genes, it is noteworthy that in *S. coelicolor*, GlnR—a major regulator for nitrogen metabolism—serves as a negative regulator for *ect* gene expression [219]. In our phylogenomic analysis (Figure 5), and in contrast to the distribution of *ectR*, we found no *glnR*- or *cosR-*type regulatory genes in close association with any ectoine/5-hydroxyectoine biosynthetic gene cluster. However, a possible genetic or physiological link of ectoine/5-hydroxyectoine biosynthesis to the overall nitrogen control in microbial cells [194] is an interesting aspect for future studies, given that ectoines are nitrogen-rich compounds (Figure 1B).

The genetic control of *ect* gene expression is embedded in the overall osmostress adjustment response of cells using the *salt-out* strategy (Figure 1A). Frequently, the size of the ectoine/5-hydroxyectoine pool is substantially reduced when other compatible solutes (e.g., glycine betaine) are imported from the growth medium. This effect can be traced through reporter fusion studies to a dampening influence of the imported solutes on the strength of *ect* transcription [116,118,206]. However, there is also a report in the literature that claims an inducing effect of imported ectoines on the transcription of the *ectABCD* gene cluster from *Streptomyces rimosus* C-2012 under salt stress conditions [220]. However, such an effect, to the best of our knowledge, has not been observed in any other microorganism in which the regulation of *ect* gene expression has been studied. 

The dampening effect of imported compatible solutes on *ect* transcription is not unique to this particular type of promoter(s), as the activity of many osmostress responsive promoters is down-regulated when externally provided compatible solutes are accumulated [84,212,213]. Hence, it seems plausible that newly synthesized ectoines will influence *ect* promoter activity when the cellular pools of these compatible solutes rise in response to increased osmotic stress. This regulatory effect might provide the cell with a homeostatic system not to wastefully overproduce ectoines when it has attained osmotic equilibrium and it might be a contributing factor to the striking linear relationship between *ect* expression and the external salinity observed in several microorganisms [116,117]. It is currently not known whether the dampening effects of imported osmostress protectants on the strength of *ect* transcription are directly exerted via an influence on the activity of RNA-polymerase or its ability to productively interact with the *ect* promoter, or whether the effects are somehow indirectly caused by the weakened osmotic stress perceived by the microbial cell [84]. 

### 6.3. Phylogenomics of ect Genes

While the EctA (l-2,4-diaminobutyrate acetyltransferase) and EctB (l-2,4-diaminobutyrate transaminase) enzymes have close paralogs related in their amino acid sequences and function in microbial biosynthetic pathways not related to ectoine biosynthesis, the ectoine synthase (EctC) can be regarded as a diagnostic enzyme for ectoine producers. However, microorganisms have been discovered, which either possess EctC-related proteins but lack the *ectAB* genes or possess solitary *ectC*-type genes in addition to a canonical *ectABC* gene cluster [93,176,221]. Hence, when EctC is used as the search query to assess the phylogenomics of microbial ectoine producers, it is critical to inspect the gene neighborhood of each retrieved *ectC* hit. Likewise, *bona fide* ectoine hydroxylases (EctD) need to be distinguished from related 2-oxoglutarate-dependent dioxygenases with different enzymatic functions, as EctD proteins are often miss-annotated in genome sequences either as proline- or phytanoyl-hydroxylases. True EctD proteins (see Section 5.2.4) can be distinguished from the other members of the non-heme Fe(II)-containing and 2-oxoglutarate-dependent dioxygenase enzyme super-family [182] by a highly conserved consensus sequence motif harboring residues critical for substrate binding and enzyme catalysis [177,184]. 

We used the Integrated Microbial Genomes and Microbiomes (IMG/M) database of the Joint Genomics Institute (JGI) of the US Department of Energy (http://img.jgi.doe.gov/cgi-bin/w/main.cgi) [222] for our new database searches to identify putative producers of ectoines, since the web-tools of this Internet portal allow a simple evaluation of the gene neighborhood of the gene(s) of interest. For our analysis, we used the amino acid sequence of the EctC protein from *V. salexigens* as the search query, since this particular ectoine synthase has been intensively characterized by both enzymatic and structural approaches [93,177]. At the time of our search (13 November 2017) the IMG/M database contained 56,624 bacterial and 1325 archaeal genomes; from this data set we identified 4493 bacterial and 20 archaeal EctC-type proteins. It should be noted that the IMG/M database, like other microbial genome databases, is skewed with respect to the types of microorganisms covered because sequences of certain microbial species/strains are strongly overrepresented. For instance, in the dataset of 4493 bacterial genomes containing *ectC*-type genes, 1215 *Vibrio* species/strains (with 443 *V. cholerae* strains alone) and 511 *Streptomyces* isolates are represented. 

When only considering fully sequenced microbial genomes, our final dataset contained 499 bacterial and 11 archaeal species/strains that collectively possessed 582 predicted EctC-type proteins. We inspected these genome sequences for the presence of other ectoine biosynthesis related genes (*ectAB*, *ectD, ask_ect, ectR*) in the neighborhood of *ectC* or elsewhere. We retrieved the 582 EctC-related protein sequences, aligned them using the MAFFT multiple amino acid sequence alignment server (https://mafft.cbrc.jp/alignment/server/) [192], and then conducted a clade analysis of the putative ectoine synthase proteins using bioinformatics resources provided by the Interactive Tree of Life (iTOL software) (https://itol.embl.de/) [193] (Figure 5). We have rooted the EctC-protein based tree with out-group sequences of several microbial cupin-type proteins [182,183] not involved in ectoine biosynthesis, as the EctC synthase belongs to this protein superfamily [176]. In Figure 5, we highlight not only the taxonomic affiliation of the microorganisms from which we retrieved the particular EctC sequence, but also the presence of the ectoine hydroxylase EctD [96,97,98], that of the specialized aspartokinases Ask_Ect [122,166,168], and that of the regulatory protein EctR [99,198,215]. Data from this analysis of the genetic configuration of ectoine/5-hydroxyectoine biosynthetic genes (*ectABC/ectD*) in 510 completely sequenced bacterial and archaeal genomes of predicted ectoine/5-hydroxyectoine producers and additional genes involved in providing the ectoine biosynthetic precursor l-aspartate-β-semialdehyde (*ask_ect*) or in the transcriptional control of *ect* gene expression (*ectR*) are summarized in Table 1.

EctC-type proteins are phylogenetically associated with ten bacterial (including five subphyla of the *Proteobacteria*) and two archaeal phyla. In this clade analysis, EctC proteins that are encoded within true *ect* gene clusters follow, in general, the taxonomic affiliation of the predicted ectoine-producing microorganism. In those few cases where this is not the case, their position in the EctC-derived protein sequence clade can probably be explained by lateral gene transfer events (Figure 5). The EctC protein tree is dominated by ectoine synthases originating from *Actinobacteria* and from *Alphaproteobacteria*, *Betaproteobacteria*, and *Gammaproteobacteria*, which together make up 91% of our dataset. EctC proteins from members of the other EctC-containing ten bacterial phyla or subphyla (*Firmicutes*, *Delta-* and *Epsilonproteobacteria, Nitrospirae, Planctomycetes, Chrysiogenetes, Deferribacteres, Chloroflexi, Cyanobacteria*, and *Spirochaetes*) are only scarcely represented (Figure 5). Because of the existing bias of available genome sequences in databases, it is too early to conclude how much of the apparent incidence of ectoine synthesis actually differs between these phyla or arises from insufficient representation of some phyla in the IMG/M database. 

Lateral gene transfer is a major driver of microbial evolution [223,224] and has in particular shaped the genome of Archaea that acquired many genes from bacterial donors [225]. This is also evident for the rare cases where EctC-type proteins have been detected in Archaea [92]. In our dataset, 11 archaeal EctC protein sequences cluster in three different locations in the tree. These genomes represent members of two archaeal phyla, the *Thaumarchaeota* and *Euryarchaeota* (Figure 5). All 11 archaeal representatives in our dataset possess a complete *ectABC* gene cluster. The EctC proteins of the three marine representatives of the *Thaumarchaeota* (all strains of *Nitrosopumilus* sp.) cluster with that of the marine bacterium *Planctomyces brasiliensis* (Figure 5). In contrast to the joint clustering of the EctC proteins from the three *Thaumarchaeota*, the eight EctC proteins from the *Euryachaeota* are present in two different segments of the phylogenomic EctC protein tree. Three EctC proteins from various *Methanobacterium formicicum* strains are part of a cluster of EctC proteins present in strictly anaerobic members of rather heterogeneous bacterial taxa that comprise representatives of the phyla *Chrysiogenetes*, *Deferribacteres*, and *Deltaproteobacteria* (Figure 5).

In our dataset, 437 microbial genomes contained the *ectC* gene in the immediate vicinity of other *ect* genes; 76 genomes contained only *ectC* (e.g., *Pseudomonas fluorescens* L228, *Burkholderia multivorans* CEPA 002), and in 11 genomes, a complete set of ectoine biosynthetic genes was present, in addition to a single orphan *ectC* (e.g., *Rhizobium gallicum*, *Mycobacterium abscessus* FLAC 0046). Another subgroup of the inspected genomes contained several orphan *ectC* genes but no complete *ect* gene cluster (31 genomes) (e.g., *Pseudomonas syringae* pv. syringae B301D, *Burkholderia cepacia*). Interestingly, some bacteria contained several complete *ect* biosynthetic gene clusters (e.g., *S. reticuli*, *Streptomyces flavogriseus, Rhodovulum sulfidophilum* DSM 1374). From this extended phylogenomic analysis, it is apparent that the vast majority (75%) of *ectC*-containing genomes contain a complete set of ectoine biosynthetic genes. Most orphan *ectC* gene products cluster close to the root of the tree, possibly indicating early evolutionary states (Figure 5). In a notable number of instances, microorganisms carrying both solitary *ectC* genes and additional *ect* gene clusters, or even several copies of complete *ect* gene clusters were detected. This leaves 76 genomes in our dataset, which contain exclusively solitary *ectC* genes.

Solitary *ectC* genes were first discovered in the context of a genome-driven investigation of compatible solute synthesis in the plant pathogen *Pseudomonas syringae* pv. syringae B728a [221]. This bacterium does not produce ectoine naturally under laboratory conditions, as it lacks the *ectAB* genes. However, when surface-sterilized leaves of its host plant *Syringa vulgaris* were added to high-salinity grown cultures, ectoine production was observed, indicating that the plant provides the substrate (*N*-γ-ADABA) (Figure 2) for the EctC ectoine synthase, and that the solitary EctC-type protein *P. syringae* pv. syringae B728a was functional [221]. Indeed, heterologous expression of the solitary *ectC* gene from *P. syringae* pv. syringae B728a in an *ectC* mutant of *H. elongata*, led to ectoine production. However, while externally provided *N*-γ-ADABA was readily imported by *P. syringae* pv. syringae B728a, the expected ectoine formation was not observed [221]. Hence, this dataset is, in its core, not yet conclusive. Previous database searches have already indicated that the existence of solitary EctC-type proteins is not an isolated incident in *P. syringae* pv. syringae B728a [92,176]; we detected their presence in 13% out of the studied 457 genomes (Table 1).

EctC-type proteins can be assigned to six major clusters of sequence similarity. The three most basal of these clusters contain most of the proteins from orphan *ectC* genes, while the three others contain all EctC proteins encoded by *ect* gene clusters and only a few by isolated genes (Figure 5). The most basal major cluster (group 1) exclusively represents EctC-like proteins from various strains of *M. abscessus,* which may not be true ectoine synthases because the same strains also contain paralogs of more conventional EctC proteins. The next two major clusters (groups 2 and 3) correspond to most other organisms containing orphan *ectC* genes and comprise mostly members of the *Alphaproteobacteria*, notable groups of *Actinobacteria*, *Betaproteobacteria* and *Gammaproteobacteria*, together with two strains affiliated with the *Cyanobacteria* and two with the *Deltaproteobacteria* (Figure 5). It is currently not clear whether the solitary EctC proteins are remnants of a previously intact ectoine biosynthetic route, whether they were recruited by the EctAB proteins to form the ectoine biosynthetic pathway as we know it today (Figure 2), or whether they have evolved a new enzymatic function that nevertheless might allow in a side-reaction the cyclization of the *N*-γ-ADABA molecule to ectoine. However, the placement of the orphan EctC protein from *P. syringae* [221] in group 2 (Figure 5) suggests that these proteins might represent catalytically competent ectoine synthases. Still, careful genetic and biochemical analysis will be required in the future to establish the true function of these solitary EctC-type proteins. 

The major group 4 contains mainly EctC proteins from *Firmicutes*, marine *Gamma-* and *Betaproteobacteria*, together with rare orthologs from the *Planctomycetes*, *Spirochaetes*, *Delta*- and *Epsilonproteobacteria*, *Chrysiogenetes*, *Chloroflexi*, *Deferribacteres* and two archaeal groups comprising the *Nitrosopumilus* and *Methanobacterium* strains. Group 5 contains the proteins from mostly marine *Alphaproteobacteria*, *Betaproteobacteria* and *Gammaproteobacteria*, including many members of the *Roseobacteriales*, *Halomonadadales* and *Vibrionales*, together with one spirochaete, one sulfate-reducing *Deltaproteobacterium*, three *Leptospirillum* strains affiliated to the *Nitrospirae* and the remaining *ect* gene clusters containing archaeal species representing five members of the *Methanosarcinales*. Finally, group 6 represents exclusively terrestrial *Actinobacteria*, with the exception of one basal EctC sequence from a strain of the alphaproteobacterium *R. gallicum* (Figure 5).

The formation of 5-hydroxyectoine depends on the prior synthesis of ectoine and is catalyzed in a position- and stereo-specific reaction by the ectoine hydroxylase (EctD) [98,177]. The *ectD* gene can be found in one of two different genetic contexts: (i) it either can be present in the vicinity of other *ect* biosynthetic genes, or (ii) it can be encoded somewhere else in the genome of a predicted ectoine producer [96,97,98]. In our dataset of 510 predicted ectoine producers, 314 (62%) possess an *ectD* gene; in 259 genomes, *ectD* is part of the biosynthetic gene cluster, and 68 *ectD* genes are found outside of the *ect* gene cluster (Figure 4 and Figure 5). Some organisms (20 genome sequences) possess an external *ectD* gene, in addition to the *ectD* gene encoded in the *ect* gene cluster. Since the EctD enzyme is a member of the non-heme-containing, iron(II)- and 2-oxoglutarate-dependend dioxygenase enzyme superfamily [177,185], all predicted 5-hydroxyectoine producers are either aerobic or, at least, oxygen-tolerant microorganisms. This can be nicely observed in those Archaea that are predicted to synthesize ectoine either alone or in combination with 5-hydroxyectoine. In the strictly anaerobic methanogenic Archaea belonging to the genera *Methanosaeta* and *Metanobacterium*, only an *ectABC* cluster can be found, while in the oxygen-dependent nitrifying Archaea of the genus *Nitrosopumilus*, *ectABCD* gene clusters are present [92] (Figure 5). 

As outlined above, some *ectABC(D)* gene clusters are associated with a gene (*ask_ect*) encoding a specialized aspartokinase [122,166,167,168]. We assessed the phylogenetic occurrence of the Ask_Ect (Figure 5) and the genetic organization of its structural gene within the context of the *ect* biosynthetic genes (Figure 4). In our dataset of 510 putative producers of ectoines, 133 ectoine/5-hydroxyectoine biosynthetic gene clusters contained the gene for the specialized aspartokinase. These gene clusters are primarily found in *Alphaproteobacteria* and *Gammaproteobacteria* (Figure 5). 

Ectoine producers can populate ecological niches with rather different attributes. This is actually not surprising, because microorganisms will experience increases in the environmental osmolarity not only in marine and high-saline surroundings (e.g., open ocean waters, marine sediments, salterns, brines), but also, for instance, when the soil slowly dries out. If one views the putative ectoine/5-hydroxyectoine producers in an ecophysiological context, many marine and terrestrial microorganisms are represented, as are some bacteria that live associated with plants or animals. Among the latter group of microbes, bacteria are found that are beneficial to plant growth (e.g., many *Rhizobium*, *Sinorhizobium* or *Bradyrhizobium* strains), others are formidable plant pathogens (e.g., many *Pseudomonas syringae* pathovars). Likewise, some of the putative ectoine/5-hydroxyectoine producers are human or animal pathogens (e.g., *V. cholerae*, *M. abscessus, B. cepacia, B. parapertussis,* or *Bordetella bronchioseptica*). Some ectoine producers are also found among microorganisms that live in rather specialized habitats. A striking example is the gammaproteobacterium *Teredinibacter turnerae*, an intracellular endosymbiont in the gills of *Lyrodus pedicellatus*, commonly known as shipworms. This mollusk digests wood immersed in salt water, a catabolic process that relies on cellulases produced by *T. turnerae* [226]. Interestingly, ectoine/5-hydroxyectoine producers are also found in a few representatives of the phylogenetically deep-branching phylum *Planctomycetes*, microorganisms with highly interesting cell biology that are widely distributed in marine and terrestrial habitats. Physiological studies with slight halophilic representative of the genus *Planctomyces*, *P. brasiliensis* (recently re-classified as *Rubinisphaera brasiliensis*) and *Planctomyces maris* (recently re-classified as *Gimesia maris*) showed that ectoine and 5-hydroxyectoine play major roles in osmostress adaptation [227]. Attesting to the metabolic flexibility of these microorganisms under severe osmotic stress conditions, non-nitrogen-containing compatible solutes (e.g., sucrose and glucosylglycerate) are produced when nitrogen becomes limiting [227]. 

Ectoine/5-hydroxyectoine producers are also found in ecosystems whose salinity is not particularly high; one example is *A. cryptum*, a heterotrophic alphaproteobacterium that thrives in acidic, metal-rich environments, but which is not known to tolerate high concentrations of salt [180]. One also needs to keep in mind that taxonomically closely related microorganisms can rely, as far as the accumulation of ectoines is concerned, on the accumulation of different types of compatible solutes. This is exemplified by studies with the marine predatory heterotrophic myxobacteria *Enhygromyxa salina* SWB007 and *Plesiocystis pacifica* SIR-1 [228]. While *P. pacifica* SIR-1 relied on the accumulation of amino acids for its osmostress adjustment process, *E. salina* SWB007 employed, besides glycine betaine, 5-hydroxyectoine as its dominant compatible solute under high-salinity growth conditions. Accordingly, no ectoine/5-hydroxyectoine biosynthetic genes were found in the genome sequence of *P. pacifica* SIR-1, while an *ect_ask-ectABCD* gene cluster was present in the genome sequence of *E. salina* SWB007. This ectoine/5-hydroxyectoine biosynthetic gene cluster is also associated with a copy of the *ectR* regulatory gene [228]. 

While the *ect* genes are widely distributed in ecophysiologically different types of microorganisms, there is evidence in certain groups of ectoine/5-hydroxyectoine producers for ecotype diversification. For instance, ectoine/5-hydroxyectoine biosynthetic genes were found not only in the archaeon *N. maritimus* strain SCM1 [92] but are also present in the draft genomes of halotolerant *Nitrosopumilus* species populating brine-seawater interfaces, whereas they are not present in genomes of *Nitrosopumilus* species enriched from low-salinity estuary and costal environments [229]. The clearest evidence reported to date for an association of ectoine biosynthesis with microbial niche diversification stems from a comprehensive phylogenomic analysis of *Rhodobacteraceae* [230]. These *Alphaproteobacteria* are metabolically highly versatile and are key players in global biogeochemical cycling [231,232]. Based upon the analysis of 106 genome sequences, Simon et al. [230] found that during the evolution of this group of microorganisms several shifts between marine and non-marine habitats occurred and signature changes in genomic content reflect the different ecosystem populated by members of the *Rhodobacteraceae*. During this process, marine *Rhodobacteraceae* gained the genes for ectoine synthesis and that for the production of the compatible solute carnitine, and they also acquired the ability to import this latter osmostress protectant [230]. In a study addressing the phylogeny of the ectoine biosynthetic genes in aerobic, moderate halophilic methylotrophic bacteria, Reshetnikov et al. [99] found that the amino acid sequence relationship of the ectoine biosynthetic proteins did not strictly correlate with the phylogenetic affiliation of the studied methylotrophic species and strains, thereby suggesting that the ability to synthesize ectoine most likely results from lateral gene transfer events. Such gene transfer events are clearly manifested when one views the position of the EctC proteins from Archaea within the clade analysis of ectoine synthases present in Bacteria (Figure 5) [92]. 

## 7. Scavenging Ectoines as Stress Protectants from Environmental Sources

Ectoines are produced and accumulated in high-osmolarity-stressed microorganisms to exceedingly high cellular levels [91,94]. They are released from these producers through the transient opening of mechanosensitive channels during osmotic down-shocks, through secretion, by decomposing microbial cells attacked by phages or toxins, or through the predatory activity of microorganisms and eukaryotic cells [233]. Hence, it is not surprising that environmentally compatible solutes, including ectoines, have been detected in different ecosystems [234,235,236,237,238,239]. As a result, the presence of cell-free ectoines provides new opportunities for microorganisms living in the same habitat as the ectoine producers by allowing them to ameliorate osmotic or temperature stress through import of these compatible solutes. 

Transport systems for compatible solutes are ubiquitous in microorganisms, and these are typically osmotically regulated both at the level of transport activity and in the transcriptional response of their structural genes [2,6,10,240,241,242]. The activity regulation of osmolyte transporters provides the cell with a practically instantaneous adjustment response to osmotic up-shift [240,242,243,244,245,246] that, depending on the severity, can strongly impair growth [247]. The transcriptional induction of the transporter genes will then provide enhanced transport capacity for osmostress protectants to permit growth under sustained osmotically unfavorable conditions [2,84,85]. Hence, uptake systems for compatible solutes [1,2,3,241], or for their biosynthetic precursors (e.g., choline for the synthesis of glycine betaine) [171,248], are integral parts of the overall osmostress adjustment strategy of many microbial cells (Figure 1A). They typically possess *K*_m_ values in the low μM range, thereby allowing the recovery of stress protective solutes from scarce environmental sources. Often, a given microbial cell possesses several osmostress protectant uptake systems, which frequently differ in their substrate profile and mode in which the transport process is energized [10,84,85,240,249], thereby providing additional flexibility to the osmotically challenged cell. 

Ectoine/5-hydroxyectoine transport systems involved in alleviating osmotic or temperature stress have been characterized in various Gram-negative and Gram-positive bacteria. These importers belong to four different transporter families: (i) binding protein-dependent ABC transporters [250,251] that use ATP to fuel substrate translocation across the cytoplasmic membrane (e.g., the ProU system from *E. coli*, the OusB system from *Erwinia chrysanthemi,* the OpuC transporter from *B. subtilis* and the ProU system from *Vibrio anguillarum*) [121,187,252,253], (ii) members of the Major Facilitator Family (MFS) [254] that are dependent on the proton motif force (e.g., the ProP and OusA system from *E. coli* and *E. chrysanthemi*, respectively) [243,255], (iii) members of the Betaine-Choline-Carnitine Transporters (BCCT) [241] that are energized either by proton or sodium gradients (e.g., the OpuD transporter from *B. subtilis,* EctT from *V. pantothenticus*, EctM from *M. halophilus,* EctP and LcoP from *C. glutamicum*) [78,256,257,258,259], and (iv) members of the periplasmic binding protein-dependent tripartite ATP independent periplasmic transporter family (TRAP-T) [160] that are energized by proton or sodium gradients (e.g., the TeaABC system from *H. elongata*) [161]. Often, transporters used for the import of ectoines exhibit broad substrate specificity (e.g., the ProU and ProP systems from *E. coli* and the OpuC transporter from *B. subtilis*) [84,85,188,189], but dedicated importers for these compounds are also known (e.g., the TeaABC system from *H. elongata* and the EctT transporter from *V. pantothenticus*) [78,161]. 

In the context of osmostress-responsive transporters for ectoine/5-hydroxyectoine, it is important to note that some compatible solute transporters (e.g., BCCT- and TRAP-types) import substantial amounts of Na^+^ into the cell, along with the stress-relieving substrate. For instance, the glycine betaine transporter BetP from *C. glutamicum*, the biochemically and structurally best-studied transporter of the BCCT family [241], to which the ectoine/5-hydroxyectoine transporter EctT, EctM, EctP, and LcoP also belong [78,257,258,259], has a stoichiometry of two Na^+^ ions per imported glycine betaine molecule [241,245,246,260]. Since substantial compatible solute pools are generated through transport, effective export systems for the co-transported cytotoxic Na^+^ ions are key players in the overall osmostress adjustment strategy of microorganisms using the *salt-out* strategy (Figure 1A).

High-resolution crystal structures of the TeaA periplasmic ligand-binding protein, in complex with either ectoine (PDB accession code 2VPN) or 5-hydroxyectoine (PDB accession code 2VPO), have been determined [261]. The crystal structure of another ectoine-binding protein (OpuCC) (PDB accession code 3PPR) has also been reported [262]. OpuCC is the extracellular solute receptor of the promiscuous, osmotically inducible OpuC ABC transporter from *B. subtilis* [84,85]. In contrast to the high affinity TeaABC system [261], OpuC imports ectoine only in a side reaction (the *K*_i_ of ectoine import via OpuC is about 1.5 mM) [252] and thus will not play a decisive role for ectoine import in natural settings of *B. subtilis* where ectoines will only be present in very low concentrations [234,235,236,237]. 

## 8. Ectoines as Nutrients

### 8.1. Physiology

A hallmark of microorganisms is their enormous metabolic potential. There is essentially no compound synthesized by microorganisms that cannot be catabolized, either by the producer cell itself or by other microorganisms living in the same habitat. This is also true for the nitrogen-rich ectoine/5-hydroxyectoine molecules (Figure 1B); their use as sole carbon, nitrogen and energy sources has been demonstrated for different microbial species [74,158,190,263,264,265,266]. Environmental ectoines have been detected in various ecosystems [234,235,236,237,239], and their presence provides new opportunities for microbial ectoine consumers living in habitats that are also populated by ectoine producers. Ectoine-catabolizing microorganisms can scavenge these valuable compounds from the environment through high-affinity, substrate-induced transport systems such as the ABC-system EhuABCD or the TRAP transporter UehABC [263,265,267,268]. Since ectoines are unlikely to be continuously present in a given habitat, it makes physiologically sense for nutrient-limited microorganisms to exert a tight transcriptional control over ectoine/5-hydroxyectoine importer and catabolic genes. We will address below the taxonomic affiliation of ectoine consumers, the catabolic route for ectoines, transport systems for their acquisition, and the genetics underlying the transcriptional control of ectoine/5-hydroxyectoine import and degradation gene clusters. 

### 8.2. Genetics and Phylogenomics of Ectoine Catabolic Genes

**Figure 6:**
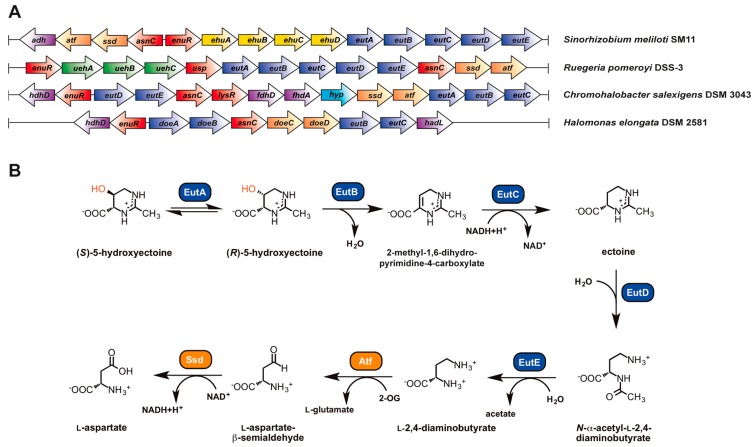
Genetics and catabolic pathways for the utilization of ectoine and 5-hydroxyectoine as nutrients. (**A**) Genetic organization of the ectoine/5-hydroxyectoine-catabolic gene cluster in *Sinorhizobium meliloti* SM11 [265], *Ruegeria pomeroyi* DSS-3 [263,270], *Halomonas elongata* DSM 258 [158] and *Chromohalobacter salexigens* DSM 3043 (predicted from the genome sequence) [274]. In addition to the transporter and catabolic genes discussed in the main text, some of these gene clusters contain genes with yet undefined roles in ectoine catabolism. Their gene products have bioinformatically predicted functions as alcohol dehydrogenase (*adh*), hydroxyacid dehydrogenase (*hdhD*), formate dehydrogenases (*fdhD*, *fdhA*), haloacid dehalogenase (*hadL*), transcriptional regulator (*lysR*) and a hypothetical protein (*hyp*). (**B**) Predicted pathway for the catabolism of ectoine and its derivative 5-hydroxyectoine in *R. pomeroyi* DSS-3. The EutABC-enzymes are predicted to convert 5-hydroxyectoine in a three-step reaction into ectoine. The ectoine ring is subsequently hydrolyzed by the EutD enzyme, resulting in the production of *N*-α-ADABA, an intermediate, which is then further catabolized to l-aspartate by the EutE, Atf and Ssd enzymes. These data were compiled from the literature [158,263,270]. The ectoine-derived metabolites *N*-α-ADABA and l-2,4-diaminobutyrate (DABA) serve as inducers for the transcriptional control of the ectoine/5-hydroxyectoine import and catabolic gene clusters by the EnuR regulatory protein [270].

While the use of ectoines as nutrients has been known about for quite some time [74,190,263,264,265,266], inroads into a molecular and biochemical understanding of ectoine/5-hydroxyectoine catabolism have only been made recently. In a pioneering study, Jebbar et al. [265] used a proteomics approach to identify proteins induced in cells of the symbiotic plant-root-associated soil bacterium *Sinorhizobium meliloti* grown in the presence of ectoine. The protein products of eight ectoine-induced genes were identified by mass-spectrometry, and their genes co-localized in the same gene cluster together with several other genes whose products had not been detected by proteomics (Figure 6A) [265]. This gene cluster is carried by the pSymB mega-plasmid of *S. meliloti*. Four of the nine ectoine-inducible genes encode the components of a binding-protein-dependent ABC-transporter (EhuABCD; *ehu*: *e*ctoine-*h*ydroxyectoine-*u*ptake) and form an operon with five additional genes (*eutABCDE; eut*: *e*ctoine *ut*ilization) predicted to encode enzymes for ectoine/5-hydroxyectoine catabolism. The entire *ehuABCD-eutABCDE* gene cluster is preceded by a gene encoding a member of the GntR superfamily of transcriptional regulators [269] (Figure 6A), a regulatory gene that is now known as *enuR* (*e*ctoine *nu*trient *r*egulator) [270]. Divergently oriented from the *S. meliloti ehuABCD-eutABCDE* operon was an additional regulatory gene (*asnC*) encoding a member of the AsnC/Lrp family of the *feast-and-famine* DNA-binding proteins [271,272,273] and three ectoine-inducible genes functionally annotated as an aminotransferase, an oxidoreductase, and a succinate semialdehyde dehydrogenase (Figure 6A) [265]. Using an *ehuAB-uidA* transcriptional reporter system, enhanced expression of the reporter fusion was observed when either ectoine or 5-hydroxyectoine was present in the growth medium, but neither glycine betaine nor high salinity triggered enhanced gene expression [265]. Hence, the *ehuABCD-eutABCDE* operon is substrate inducible, as expected for a catabolic system. Building on these findings in *S. meliloti* [265], related ectoine/5-hydroxyectoine import and catabolic gene clusters were identified and experimentally studied in *H. elongata* [158] and the marine bacterium *Ruegeria pomeroyi* DSS-3 [263]. For *H. elongata* [158], a genetic nomenclature different from those used for the annotation of the ectoine/5-hydroxyectoine catabolic genes in *S. meliloti* and *R. pomeroyi* was used; in Figure 6A we have compared the corresponding gene organization in these three organisms to minimize confusion that can be caused by the different annotation of the *H. elongata* genes. 

In this figure, we have also included the genetic organization of the ectoine catabolic genes from *C. salexigens*, a gammaproteobacterium taxonomically closely related to *H. elongata*, in which ectoine/5-hydroxyectoine synthesis has been studied in quite some detail [80,194] and in which catabolism of these compounds has also been physiologically assessed [266]. An inspection of the ectoine/5-hydroxyectoine catabolic and importer gene clusters from these four organisms reveals a considerable variation in genetic organization and gene content (Figure 6A). For instance, while the *S. meliloti* gene cluster encodes an ABC import system (EhuABCD) for ectoines [265,267], that of *R. pomeroyi* DSS-3 possesses a TRAP transporter (UehABC) for their uptake [263,268]. In contrast, the *H. elongata* and *C. salexigens* catabolic gene clusters lack genes for an import system for ectoines altogether (Figure 6A), but they both possess genes for UehABC-related ectoine/5-hydroxyectoine-specific import systems (TeaABC) [161,261] somewhere else in their genomes [263,270]. It is, however, not clear whether the TeaABC transporter serves for the acquisition of ectoines as nutrients since the transcription of the *teaABC* operon is osmotically inducible in *H. elongata* [161]. 

Using the ectoine hydrolase (EutD), a key enzyme of ectoine catabolism (Figure 6B) [158,263], as a search query for the analysis of 32,523 bacterial and 654 archaeal genomes, 539 EutD orthologes were found [263]. Inspection of the *eutD* gene neighborhoods then revealed a diverse genetic organization and gene content of the ectoine/5-hydroxyectoine import and catabolic gene clusters on a broad scale [158,263]. This stands in contrast to the rather stable and evolutionarily conserved genetic organization of the ectoine/5-hydroxyectoine biosynthetic genes as an *ectABC/ectD*-type operon (Figure 4A). Strikingly, while microbial ectoine/5-hydroxyectoine producers can be found in ten bacterial and two archaeal phyla (Figure 5), ectoine consumers are taxonomically restricted to the phylum of *Proteobacteria* [263]. In the particular dataset of the 539 *eutD*-containing microbial genomes inspected by Schulz et al. [263], 58% belong to the *Alphaproteobacteria*, 15% were from *Betaproteobacteria,* 27% were from *Gammaproteobacteria*, and there was only a single representative (*Desulfovibrio bastinii* DSM 16055) from the *Deltaproteobacteria*. 

Interestingly, among the 539 predicted ectoine/5-hydroxyectoine consumers, 100 microorganisms are predicted to synthesize ectoines as well [263]. The simultaneous presence of ectoine/5-hydroxyectoine biosynthetic and catabolic genes in a given microorganism will require a careful genetic and physiological wiring of these physiologically and biochemically conflicting processes (see Section 7.5) in order to avoid a futile cycle (see Section 7.5). One of the organisms capable of ectoine synthesis and degradation is *H. elongata*, and in the context of its genome annotation and analysis of ectoine synthesis and catabolism, Schwibbert et al. [158] suggested that the ability to both synthesize and degrade ectoines might aid the *H. elongata* cell to physiologically navigate osmotic downshifts. If this hypothesis holds true, then it can only apply to situations where the environmental osmolarity is decreased rather slowly, since mechanosensitive channels (genes for these safety-valves are present in *H. elongata*) (Figure 1A) will otherwise open within milliseconds during harsh osmotic downshifts to reduce the ectoine pool rapidly [11]. Furthermore, Schwibbert et al. [158] calculated that a futile cycle of simultaneous synthesis and degradation of ectoine would saddle the metabolism of *H. elongata* under already energetically and physiologically challenging osmotic conditions [37,106,197] with the expenditure of two additional ATP molecules per turn of ectoine synthesis. Microorganisms capable of both ectoine synthesis and catabolism are quite prevalent in nature; in the dataset of Schulz et al. [263], about 19% of ectoine/5-hydroxyectoine producers were also able to degrade these compounds. It will thus be of considerable interest to learn in future studies how these types of microbes can avoid wasteful futile cycles under steady-state high osmolarity growth conditions. 

### 8.3. Transporters for the Scavenging of Ectoines for Their Use as Nutrients

Ectoines present in the environment occur at very low concentrations [234,235,236,237,238,239]; hence, high-affinity transporters are required for their recovery and use as nutrients. The EhuABCD ABC transporter from *S. meliloti* and the UehABC TRAP transporter from *R. pomeroyi* are uptake systems whose main function is the scavenging of ectoines for nutritional purposes [263,265,267,268]. The transcription of the underlying structural genes is substrate inducible, but they are not osmotically induced. Although the Ehu and Ueh systems belong to different transporter families (ABC and TRAP transporters, respectively) [160,250,251], they are both dependent on a periplasmic substrate-binding protein (EhuB and UehA, respectively) [267,268]. These binding proteins trap ectoines that have passed the outer membrane via passive diffusion (probably via general porins) with high affinity in the periplasm and deliver them to the core components of the Ehu and Ueh transporters present in the inner membrane for energy-dependent translocation into the cytoplasm. Ligand-binding studies with the purified EhuB and UehA proteins revealed their high affinity for ectoine and 5-hydroxyectoine; EhuB has apparent *K*_d_ values of 1.6 μM for ectoine and 0.5 μM for 5-hydroxyectoine [265,267], while UehA exhibits apparent *K*_d_ values of 1.4 μM for ectoine and 1.1 μM for 5-hydroxyectoine [268]. Crystallographic studies of the EhuB (PDB accession codes 2Q88 and 2Q89) [267] and UehA (PDB accession code 3FXB) [268] proteins in complex with ectoines revealed the details of the architecture of a ligand-binding site for these compatible solutes, thereby providing further insights into the structural principles of substrate recognition and binding of organic osmolytes that are preferentially excluded from protein surfaces [60,61,62]. 

Similar design principles for trapping the ectoine ligand were observed in the crystal structure of the binding protein (TeaA) of the TeaABC TRAP transporter from *H. elongata* [261], a system that primarily serves for the acquisition of ectoines when they are used as osmostress protectants and as a recovery system for newly synthesized ectoines that are leaked or actively excreted from the *H. elongata* producer cell [161]. Crystal structures of the TeaA protein in complex with either ectoine (PDB accession code 2VPN) or 5-hydroxyectoine (PDB accession code 2VPO) have been determined [261]. This protein has *K*_d_ values of 0.2 μM for ectoine and 3.8 μM for 5-hydroxyectoine, respectively [261]. Interestingly, the crystal structures of the UehA and TeaA ligand-binding sites are virtually superimposable [261,268], despite the fact that the TeaABC and UehABC TRAP-type transporters serve different physiological functions. Hence, nature has taken a proven transporter module for the import of ectoines and endowed the transcription of the underlying structural genes with regulatory patterns that allow the transporter either to serve in osmostress protection (TeaABC) [161], or to enable the feeding on ectoines (UehABC) [263,270]. 

As indicated above, the TeaABC-type transporter might not only serve in osmostress protection, but might also function in the acquisition of ectoines as nutrients. While genes for Ehu-type (370 genomes out of a dataset of 539 ectoine degraders) and Ueh-type (48 genomes out of a dataset of 539 ectoine degraders) transporters are widely affiliated with the corresponding catabolic gene clusters, there is a substantial group of ectoine consumers (122 representatives) that lack transporter genes in the immediate vicinity of the catabolic gene cluster [263]. Since ectoines need to be imported before they can be consumed, it is obvious that transporter genes for these compounds must be encoded somewhere else in the genomes. Perhaps additional transporters for the acquisition of ectoines as nutrients might await discovery. Notably, a sub-group (23 representatives) of predicted ectoine consumers lacking genes for transporters in the vicinity of the catabolic genes possesses genes for TeaABC-type transporters somewhere else in their genome sequence [263], and *H. elongata* is a representative of this group [158,161]. Because mutants with inactivated *ectABC* and *teaABC* genes are available [94,158,161], *H. elongata* would be well-suited to testing the idea [263] that the osmoregulated TeaABC-type transporter might also be involved in the uptake of ectoines when these are consumed. 

### 8.4. Biochemistry of Ectoine/5-Hydroxyectoine Catabolism

Building on the data reported by Jebbar et al. [265] on the identification of ectoine-inducible proteins in *S. meliloti*, Schwibbert et al. [158] made the first concrete proposal for the degradation pathway of ectoine using the blueprint of the *H. elongata* genome sequence. According to this proposal, ectoine degradation begins with the enzymatic opening of the ectoine ring by the ectoine hydrolase (DoeA/EutD; EC 3.5.4.44) to form *N*-α-acetyl-l-2,4-diaminobutyrate (*N*-α-ADABA) as a key intermediate which is further catabolized by the *N*-α-acetyl-l-2,4-diaminobutyrate deacetylase (DoeB/EutE; EC 3.5.1.125) to acetate and DABA. The DoeD/Atf enzyme then converts DABA to l-aspartate-β-semialdehyde and l-glutamate by a transamination reaction; this enzyme belongs to the family of acetyl ornithine aminotransferases. The DoeC/Ssd enzyme then further oxidizes the l-aspartate-β-semialdehyde formed by the DoeD/Atf enzyme to l-aspartate, an important intermediate in central metabolism; the DoeC/Ssd protein is an enzyme related to known succinate semialdehyde dehydrogenases (Figure 6B). Notably, this proposal for ectoine catabolism [158,263] traces the ectoine biosynthetic route (Figure 2) backwards but the types of enzymes involved in the anabolic and catabolic routes are obviously different. 

Heterologous expression of the *H. elongata* ectoine hydrolase (DoeA/EutD) in *E. coli* showed that it converts ectoine into both the alpha- and gamma-isomers of ADABA in a 2:1 ratio [158], with *N*-γ-ADABA being the main substrate for the ectoine synthase EctC (Figure 2). Since *N*-γ-ADABA does not seem to be a substrate for the DoeB/EutE enzyme (Figure 6B) [158], it is currently not clear if the formation of *N*-α-ADABA and *N*-γ-ADABA by the ectoine hydrolase (DoeA/EutD) is a specific feature of those microorganisms capable of both synthesizing and catabolizing ectoine (note that *H. elongata* possesses both pathways [158]). Otherwise the formation of *N*-γ-ADABA by the ectoine hydrolase could be rather wasteful, unless the EutE enzyme (Figure 6B) is able to transform both *N*-α-ADABA and *N*-γ-ADABA into DABA. 

Examining the ectoine/5-hydroxyectoine catabolic pathway in *R. pomeroyi* DSS-3, Schulz et al. [263] concurred with the proposal by Schwibbert et al. [158] with respect to the degradation route of ectoine to l-aspartate, but they additionally made a proposal for the conversion of 5-hydroxyectoine into ectoine. The removal of the 5-hydroxyl group from the ectoine ring is envisioned as a three-step enzymatic process that involves the EutABC proteins (Figure 6B). The first step in this reaction is the steric inversion of the hydroxy group by the racemase EutA, converting the native (*S*)-5-hydroxyectoine conformation to the (*R*)-5-hydroxyectoine enantiomer to fit the stereochemical requirements of the next enzyme, EutB (Figure 6B). The EutB enzyme belongs to the family of threonine dehydratases and might be a pyridoxal-5′-phosphate (PLP) dependent enzyme which eliminates a water molecule from the 5-(*R*)-hydroxyectoine enantiomer. The predicted reaction product of EutB is 2-methyl-1,6-dihydropyrimidine-4-carboxylate, which is proposed to be reduced to ectoine by the EutC enzyme, a protein that is thought to serve in a NADH-dependent reduction as an ectoine dehydrogenase (Figure 6B) [263]. 

We stress here that the envisioned conversion of 5-hydroxectoine to ectoine and its further catabolism to l-aspartate as suggested by Schwibbert et al. [158] and Schulz et al. [263] have not been biochemically evaluated, with the exception of the preliminary assessment of the opening of the ectoine ring by the ectoine hydrolase from *H. elongata* in cells of a heterologous host bacterium [158]. Furthermore, the rather varied gene content of ectoine/5-hydroxyectoine catabolic gene clusters (Figure 6A) [158,263] suggests that variations of the 5-hydroxyectoine to ectoine to l-aspartate catabolic route are likely to exist in microorganisms. In particular, many of these gene clusters lack a homolog of the *eutA* gene. It is also possible that some microorganisms can catabolize ectoine, but cannot use 5-hydroxyectoine as a nutrient, as suggested by the inspection of the gene content of a substantial number of ectoine catabolic gene clusters [263]. 

### 8.5. Genetic Regulation of Ectoine/5-Hydroxyectoine Catabolism

As expected for a catabolic system, the ectoine/5-hydroxyectoine import and catabolic gene clusters of *S. meliloti* and of *R. pomeroyi* DSS-3 are substrate inducible [263,265,268,270]. Detailed genetic studies with this system in *R. pomeroyi* DSS-3 revealed that an external supply of either ectoine or 5-hydroxyectoine triggers enhanced import of these compounds and strongly increases the transcription of the *uehABC-usp-eutABCDE-asnC-ssd-atf* gene cluster, forming a 13.5 Kbp poly-cistronic mRNA [268,270]. However, neither ectoine nor 5-hydroxyectoine serve as the true inducers for the de-repression of the transcription of this operon; instead two intermediates in ectoine catabolism, *N*-α-ADABA and DABA (Figure 6B), serve as the physiologically relevant inducers [270]. These compounds are recognized by EnuR, a member of the MocR/GabR sub-group of the large GntR superfamily of transcriptional regulators [275,276]. The *enuR* structural gene (*enuR*: *e*ctoine *n*utrient *u*tilization *r*egulator) is positioned upstream of the *uehABC-usp-eutABCDE-asnC-ssd-atf* gene cluster (Figure 6A) and is expressed from a separate non-ectoine responsive promoter in *R. pomeroyi* DSS-3 [270]. This situation is apparently different from that observed in *S. meliloti* where substrate induction of *enuR* transcription was reported [277]. The EnuR protein appears to play an important role in controlling the transcription of ectoine/5-hydroxyectoine import and catabolic gene clusters in many microorganisms. In the dataset of 539 putative microbial ectoine consumers analyzed by Schulz et al. [270], 456 ectoine/5-hydroxyectoine catabolic gene clusters were associated with an *enuR* gene. 

MocR/GabR-type transcriptional regulators are widely distributed in microorganisms [275,276], but are clearly an understudied sub-group of the GntR super-family [269]. The best-studied representative of the MocR/GabR group is the GabR regulator from *B. subtilis* that serves to control genes involved in the metabolism of γ-aminobutyrate (GABA) [278]. The GabR protein is a head-to-tail swapped dimer with an N-terminal DNA reading head containing a winged helix-turn-helix DNA binding motif that is connected via a long flexible linker region to a large carboxy-terminal effector binding/dimerization domain [279]. This latter domain, structurally related to aminotransferases of type-1 fold, contains a covalently bound PLP molecule. However, the C-terminal domain of GabR does not perform a full aminotransferase reaction; instead, a partial aminotransferase reaction occurs [279,280,281,282,283]. In this chemical sequence of events, the co-factor PLP binds to the side-chain of a particular Lys residue of GabR, yielding a Schiff base and thereby resulting in the formation of an internal aldimine [173]. Subsequently, the system-specific low-molecular mass effector molecule GABA binds to the PLP molecule, which then leads to the detachment of the PLP molecule from the Lys residue of GabR and the formation of an PLP:GABA complex, the external aldimine [173,279,280,281,282]. This sequence of events triggers a conformational change of the GabR dimer [279,282], which in turn dictates the DNA-binding activity of the regulatory protein to function either as an activator of the *gabTD* metabolic operon, or as a repressor of its own structural gene (*gabR*) [278]. 

**Figure 7:**
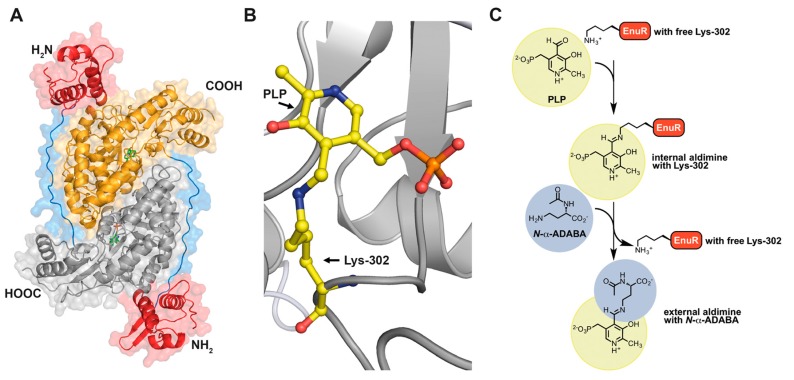
EnuR, a PLP-containing transcriptional regulator of ectoine/5-hydroxyectoine gene clusters. (**A**) in silico model of the predicted *Ruegeria pomeroyi* DSS-3 EnuR dimer that was derived from the crystallographic structure of the *Bacillus subtilis* GabR (PDB accession code 4N0B) [279]. The EnuR model was built with the SWISS-MODEL web server (https://swissmodel.expasy.org/) [286] and visualized using the PyMOL Molecular Graphics System suit (https://pymol.org/2/) [287]. The C-terminal aminotransferase-domains of the EnuR dimer are shown in grey/yellow, the N-terminal DNA-binding domains are represented in red and the flexible linkers connecting these domains are depicted in blue. Each monomer contains a PLP molecule covalently bound via an Schiff base to Lys^302^ in the aminotransferase domain [263,270]. This internal aldimine [173] is depicted in (**B**) in a close-up view. (**C**) Model for the chemistry underlying binding and release of the inducer *N*-α-ADABA to the PLP-cofactor bound to Lys^302^ of the EnuR regulator. In the first step, PLP is covalently bound by the side-chain of Lys^302^ and thus forms an internal aldimine [173]. Upon binding of the inducer *N*-α-ADABA to PLP, PLP is released from Lys^302^ and an external aldimine [173] is formed. This sequence of events is envisioned to trigger a conformational change in EnuR, thereby altering its DNA-binding properties. This scheme for inducer binding by EnuR is based upon detailed biochemical and structural analysis of the *B. subtilis* GabR regulator that uses GABA as its inducer [279,280,281,282,283].

A homology model of the EnuR dimer based on the crystal structure from the *B. subtilis* GabR regulatory protein [279] is shown in Figure 7A. The EnuR protein from *R. pomeroyi* DSS-3, as heterologously produced (in *E. coli*) and purified by affinity chromatography, has a striking yellow color [263] and possesses spectroscopic properties resembling those of PLP-containing enzymes [173,284,285]. Modeling studies identified Lys^302^ in the EnuR aminotransferase domain as the PLP-binding residue. Its substitution by a His residue (EnuR*) via site-directed mutagenesis leads to loss of the yellow color exhibited by the EnuR wild-type protein in solution and alters its authentic spectroscopic properties. When the enuR* gene was expressed in a *R. pomeroyi* DSS-3 wild-type strain (*enuR*^+^), the EnuR* protein conferred a dominant negative phenotype. In other words: the EnuR* protein abrogated the ability of *R. pomeroyi* DSS-3 to use ectoines as nutrients, since its DNA-binding to the cognate operator sequence cannot be relieved in vivo [270]. These combined genetic and biochemical data unambiguously show that the PLP molecule covalently attached to Lys^302^ is critical for the regulatory function of EnuR. EnuR acts as a repressor for the ectoine/5-hydroxyectoine uptake and catabolic genes of *R. pomeroyi* DSS-3 and *S. meliloti* since an *enuR* gene disruption mutation leads to de-repression of the corresponding gene clusters [270,277]. However, since some MocR/GabR-type transcriptional regulators can act both as repressors and activators [278], it remains to be seen in future studies if EnuR possesses these two types of regulatory attributes as well. Operator sequences for EnuR-type proteins have been deduced through bioinformatics in many microorganisms [276] and DNA fragments of *R. pomeroyi* DSS-3 and *S. meliloti* containing these in silico predicted sequences are recognized and stably bound by purified EnuR proteins from the corresponding bacteria [270,277]. In DNA-band-shift assays with the EnuR protein from *R. pomeroyi* DSS-3, specific DNA:EnuR complexes began to form at concentrations of EnuR as low as 75 nM [270]. 

The chemistry underlying the reaction between the Lys-bound PLP cofactor in MocR/GabR-type regulators and the system-specific inducer requires a primary amino group [278,279,282,283]. Although an external supply of ectoine or 5-hydroxyectoine induces the transcription of the ectoine/5-hydroxyectoine uptake and catabolic gene cluster [263,270], neither of these compounds possesses such a primary amino group (Figure 1B). Consequently, the purified and PLP-containing EnuR protein from *R. pomeroyi* DSS-3 did not bind these two ectoines [270].

It seemed logical that the system-specific inducer molecule that will interact with the Lys^302^ bound PLP co-factor is generated through the metabolism of ectoines. Indeed, several ectoine-derived metabolites possess primary amino groups (Figure 6B). Microscale thermophoresis (MST) experiments revealed that *N*-α-ADABA serves as the primary system-specific inducer for EnuR; it is bound by the EnuR-PLP protein with a *K*_d_ value of about 1.7 μM. In Figure 7C we provide a scheme for the binding of the PLP molecule to EnuR/Lys^302^ to form the internal aldemine, the subsequent reaction of the inducer *N*-α-ADABA with the covalently bound PLP molecule and the subsequent formation of the PLP:*N*-α-ADABA complex, the external aldimine [270]. Additional binding studies showed that DABA also interacts with the purified EnuR protein in a Lys^302^- and PLP-dependent fashion, but the binding constant (*K*_d_ about 457 μM) for this reaction is about 270-fold reduced in comparison with the *K*_d_ value of *N*-α-ADABA [270]. As a consequence, substantial DABA concentrations (30 mM) were required to displace in vitro the EnuR protein (also referred to in the literature as EhuR or EutR [276,277]) in DNA band-shift assays from its DNA target sequence at the ectoine/5-hydroxyectoine gene cluster of *S. meliloti* [277]. 

Apart from the high affinity of EnuR for *N*-α-ADABA, this compound has the additional advantage of being an ectoine-catabolism-specific metabolite (Figure 6B), whereas DABA also occurs as an intermediate in other metabolic and biosynthetic processes in microorganisms, including the biosynthesis of ectoine (Figure 2). Taken together, the pairing of the EnuR repressor with its covalently attached PLP co-factor and ectoine-derived metabolites (*N*-α-ADABA and DABA) establishes a sensitive intracellular trigger to relieve EnuR-mediated repression of the ectoine/5-hydroxyectoine catabolic gene cluster [270]. The finding that the isomer of the inducer *N*-α-ADABA, *N*-γ-ADABA, the main substrate for the ectoine synthase (Figure 2), is not recognized by the PLP-bound EnuR regulatory protein [270] is a physiologically highly relevant result. It is of critical importance for the group of microorganisms that are capable of both ectoine synthesis and catabolism in order to avoid a wasteful futile cycle. However, the report by Schwibbert et al. [158] that the ectoine hydrolase of *H. elongata* can generate both *N*-α- and *N*-γ-ADABA molecules raises questions about the ability of microorganisms to establish a strict genetic separation of ectoine synthesis and catabolic pathways. 

Many ectoine/5-hydroxyectoine uptake and catabolic gene clusters (494 representatives from a dataset of 539 ectoine consumers [263,270]) contain an *asnC* gene. It encodes a member of the broadly distributed AsnC/Lrp-family of transcriptional regulators that can wrap DNA into nucleosome-like structures and frequently respond in their DNA-binding properties to low-molecular mass effector molecules generated through metabolism (e.g., amino acids) [271,272,288]. In many cases, these proteins respond to feast-and-famine situations, and thereby permit the efficient exploitation of sudden burst in the supply of nutrients. Studies with *asnC* mutants from the ectoine/5-hydroxyectoine uptake and catabolic gene cluster of *R. pomeroyi* DSS-3 revealed a clear activating influence on the transcription of this operon and the ability of *R. pomeroyi* DSS-3 to use ectoine as sole carbon source was abolished in the *asnC* mutant strain [270]. It is currently unclear as to which metabolite or cellular cue AsnC responds, but given the reported data for the effector molecules of EnuR (*N*-α-ADABA and DABA) [270], we would not be surprised if this regulator uses intermediates or end-products of ectoine degradation (Figure 6B) to alter its DNA-binding activity. Preliminary DNA-binding studies with the AsnC homolog (referred to as DoeX) from *H. elongata* showed that it binds to DNA segments in the proposed regulatory region of the ectoine catabolic gene cluster [158]. Relevant for an understanding of the role played by AsnC is the fact that feast-and-famine type DNA-binding proteins can work in concert with other regulatory proteins [289], a facet in gene regulation that is probably highly relevant for the large group of microbial ectoine consumers that possess both EnuR and AsnC (85% of the 539 predicted ectoine consumers in the dataset of Schulz et al. [263,270]). 

Two-component regulatory systems (TCS) are major sensor devices through which microbial cells monitor either extra- or intracellular changes [290]. Most TCS consist of a cytoplasmic membrane-embedded histidine kinase and a cytoplasmic response regulator. Upon detection of a specific signal, the histidine kinase auto-phosphorylates using ATP as phosphor donor; it then transfers the phosphoryl group to the response regulator, which will communicate with the transcriptional apparatus of the cell to alter, in many cases, gene expression [290]. Transposon mutagenesis of *R. pomeroyi* DSS-3 revealed the involvement of such a system, NtrYX [291], in the genetic control of its ectoine/5-hydroxyectoine catabolic gene cluster [270]. The NtrX response regulator is an unusual member of the NtrC-family and its cognate sensor kinase NtrY is a protein with four predicted transmembrane regions and a large, 161-amino-acids-long, extra-cytoplasmic domain. The NtrYX TCS has been implicated in a variety of cellular functions in various microorganisms, including the control of catabolic genes for nitrogen-containing compounds [291,292,293]. Genetic inactivation of *R. pomeroyi* DSS-3 *ntrYX* genes renders this bacterium unable to use ectoine as the sole carbon source [270]. Hence, the NtrYX TCS functions as a positive regulatory device for ectoine catabolism. However, it is currently unknown whether the NtrY sensor kinase recognizes externally provided ectoine directly and what the target sequences for the NtrX response regulator in the large *uehABC-usp-eutABCDE-asnC-ssd-atf* operon are. While *enuR* and *asnC* genes are widely distributed among all branches of ectoine degrading *Proteobacteria*, the *ntrYX* genes are only found in ectoine-consuming members of the *Alphaproteobacteria* [263,270]. 

Genetic studies addressing the transcriptional regulation of ectoine/5-hydroxyectoine uptake and catabolism in the marine proteobacterium *R. pomeroyi* DSS-3 have significantly advanced the understanding of the genetic wiring of this process [270]. In the dataset reported by Schulz et al. [263,270], 45% of the 539 inspected genome sequences of predicted ectoine consumers possess all three regulatory systems (EnuR, AsnC, NtrYX) that we have described in some detail in this overview. Hence, it is highly likely that their intricate interplay will set the genetic regulation of ectoine/5-hydroxyectoine catabolism in many different microbial species and strains. On the other hand, the report by Schulz et al. [270] also revealed considerable variations in terms of the presence of the *enuR*, *asnC*, and *ntrYX* genes in a given bacterium, suggesting that variants of the regulatory circuit discovered in *R. pomeroyi* DSS-3 exist. 

While these studies already paint a rather complex picture of the genetic control of microbial ectoine/5-hydroxyectoine catabolism [270], the recent discovery of a regulatory small trans-acting RNA controlling ectoine catabolic genes in the *S. meliloti* strain Sm2B3001 [294] already adds a new dimension to this process. Transcription of the gene for this small non-coding RNA (NfeR1; Nodule formation efficiency RNA) is stimulated by high osmolarity, and lack of the NfeR1 RNA altered the expression of an array of salt-responsive genes in this symbiotic bacterium. Notably, under high-salinity growth conditions, the level of the *eutAED* mRNA is down-regulated in a NfeR1 RNA-dependent fashion [294]. However, the details of this interesting regulatory circuit, its physiological consequences, and its possible wider occurrence in ectoine-consuming microorganisms need to be further explored. 

## 9. Ectoines in Eukarya: A Recent Discovery

Ectoines have so far been considered as compatible solutes exclusively synthesized and used as stress protectants by members of the Bacteria, and by a few Archaea [91,92,93,94]. Recent studies with halophilic protists now change this picture substantially [104], since ectoine/5-hydroxyectoine biosynthetic genes have been detected in *Halocafeteria seosinensis* [100,101] and ectoine production has been directly observed in *Schmidingerothrix salinarum* [102]. *H. seosinensis* is a heterotrophic, borderline extreme halophilic nano-flagelate that actively ingests bacteria as its food source; 18S rRNA-based phylogenetic analysis placed this protist into the stramenophile linage, and it was taxonomically positioned into the order *Bicosoecida* [295]. *S. salinarum* is a bacteriovorous heterotroph as well; it is a halotolerant ciliate and a member of the order *Stichotrichia* [296]. 

Marine and hypersaline habitats are populated not only by a physiologically and taxonomically diverse group of Bacteria and Archaea [23,297] but halophilic protists are also ecophysiologically critical inhabitants of these challenging ecosystems [103]. These unicellular eukaryotes serve crucial roles as primary producers and decomposers in these habitats, and some of them exert a major influence on the abundance of microorganisms and the release of bacteria-derived metabolites into the environment through their bacterivorous activity. However, their salt-stress adaptation strategy has largely been neglected [103]. 

In their studies on the genome sequence of *H. seosinensis* and its salt-stress-responsive transcriptional profile, Harding et al. [100,101] discovered the presence and the salt-stress responsive induction of ectoine/5-hydroxyectoine biosynthetic and *ask_ect* genes. Since *H. seosinensis* is a heterotroph feeding on microorganisms living in its habitat, the detection of DNA sequences related to microbial genes is at least initially of some concern. The misinterpretation of these sequences as being of eukaryotic origin can seriously compromise assembly into DNA scaffolds of the eukaryotic genome sequence and the interpretation of biological findings [298]. In the case of *H. seosinensis*, at least for the *ectABCD* and *ask_ect* genes, one can exclude this complication, since each of these genes harbors spliceosomal introns [100,101], genetic elements that are not found in Bacteria and Archaea [299]. The *H. seosinensis* ectoine/5-hydroxyectoine biosynthetic enzymes possess N-terminal mitochondrial targeting signals, while their bacterial and archaeal counterparts are all cytoplasmic enzymes [165]. This observation suggests that the production of these compatible solutes might occur in the mitochondria of the protists, cell compartments in which the biosynthetic precursors (Glu and Asp) of ectoines are synthesized using intermediates of the Krebs cycle [101]. 

In extended database searches of eukaryotic genomes, Harding et al. [101] discovered *ectA*- and *ectC*-related sequences in previously reported transcriptional profiles of other protists and in various other Eukarya. This includes even the deuterostome animals *Branchiostoma floridae* and *Saccoglossus kowalevskii*. *B. floridae* is a lancelet, modern survivors of an ancient chordate lineage [300], while *S. kowalevskii* belongs to the hemicordate phylum, marine invertebrates that are taxonomically classified together with the Chordata as Deuterostomia [301]. Experimental proof that the protist *H. seosinensis*, or for that matter any other *ect* gene-containing eukaryote, actually produces ectoines is missing in the interesting report of Harding et al. [101]. This important gap has now been closed by a comprehensive study conducted by Weinisch et al. [102], in which the salt-stress-dependent synthesis of ectoine was directly demonstrated by ^1^H-NMR spectroscopy in the halophilic heterotrophic ciliate *S. salinarum*. Since no genome sequence of *S. salinarum* is currently publicly available, the genetic organization of the ectoine biosynthetic genes remains to be determined. Interestingly, *S. salinarum* is also able to import ectoine and can derive osmostress protection from this process [102]. 

Detailed phylogenetic considerations reported by Harding et al. [100,101] on the *H. seosinensis ectABC-ectD* genes lead to the conclusion that they might have been acquired via lateral gene transfer from a prokaryote and were subsequently genetically adjusted to the transcriptional and translational apparatus of the new eukaryotic host. Considering that both *H. seosinensis* and *S. salinarum* are predatory protists [103], this is a plausible evolutionary scenario, particularly since many microbial ectoine/5-hydroxyectoine producers are inhabitants of high-saline ecosystems (Figure 5) [91,92,93,94]. It is well established that Eukarya can acquire novel metabolic traits and stress resistance determinants by stealing pre-formed gene clusters from microorganisms [302]. Since ectoines are potent protectants against osmotic, desiccation, and temperature stress, it is highly likely that the acquisition of *ect* genes by *H. seosinensis* and *S. salinarum* from their microbial food prey [100,101,104] will provide a distinct growth and survival advantage to these eukaryotic cells in their physiologically challenging high-salinity habitats [103]. 

Another interesting finding related to the synthesis of ectoines by Eukarya stems from a recent study by Landa et al. [303], in which the remodeling of the transcriptional profile of *R. pomeroyi* DSS-3 co-cultured with the diatom *Thalassiosira pseudonana* was assessed. The observed gene expression pattern indicates that, in addition to dihydroxypropanesulfonate, xylose, and glycolate, ectoine also fueled carbon and energy metabolism of the heterotroph *R. pomeroyi*. In view of the findings on the substrate-induction of the ectoine/5-hydroxyectoine uptake and catabolic genes [263,265,270], the report by Landa et al. [303] implies that the diatom produces and releases ectoine/5-hydroxyectoine that are then detected by the prokaryotic partner and exploited as a nutrient [263,270]. This interpretation rests on the assumption that the culture of *T. pseudonana* used in this study [303] is truly axenic. The finding of Landa et al. [303] and the genetic data on the transcriptional induction of the ectoine/5-hydroxyectoine uptake and catabolic gene cluster by intermediates in ectoine degradation (Figure 6 and Figure 7) [263,270] have broader implications. Members of the metabolically and ecophysiologically versatile *Roseobacter* clade are not only found as widespread free-living members of marine habitats, but also associate closely with the cells of diverse phytoplankton groups in the ocean [230,232]. Hence ectoines could play an important role in establishing and maintaining ecophysiological relevant food webs in various ecological niches. 

## 10. Conclusions and Perspectives

The data presented here provide the most comprehensive study to date on the phylogenomics of the ectoine (*ectABC*) and 5-hydroxyectoine (*ectD*) biosynthetic gene clusters, and the genes functionally associated with them, with respect to the production (*ask_ect*) of their biosynthetic precursor or the transcriptional regulation (*ectR*) of their structural genes (Figure 5). This data set can therefore serve as a reference point for the distribution of the *ect* genes in future studies, as new genome sequences of Bacteria and Archaea are determined at an ever-increasing pace. Despite the existing bias of the available genome sequences in databases, one can conclude from our phylogenomic analysis that the taxonomic affiliation of presumed ectoine/5-hydroxyectoine producers is dominated by representatives of the *Actinobacteria* and members from the *Alphaproteobacteria*, *Betaproteobacteria*, and *Gammaproteobacteria*, which together make up 91% of our dataset. Although some ectoine/5-hydroxyectoine producers are found among members of the Archaea, we conclude that the synthesis of ectoines is primarily a bacterial trait, since available evidence (Figure 5) points to the transmission of the *ectABC/ectD* genes via lateral gene transfer into the genomes of a restricted number of Archaea from members of the Bacteria [92]. This evolutionarily important process [223,224,225,302] is in all likelihood also responsible for the acquisition of *ect* biosynthetic genes by unicellular Eukarya that live in high-saline habitats [100,101,102]. This recent finding opens new avenues of research, and follow-up studies might hold surprising discoveries. Our overview on the phylogenomics of *ectABC* and *ectD* biosynthetic genes can also aid the further development and biotechnological exploitation of natural and synthetic microbial cell factories for ectoines, commercially high-value natural products [86,91,94,145,146,148], since there are many microorganisms with different life-styles to choose from (Figure 5).

The accumulation of ectoines by high-osmolarity/salinity stressed cells through synthesis and import [1] has a major influence on the hydration status of the cytoplasm, and hence on cell volume and turgor [2,10,108,109,110,111] and their function-preserving attributes [91,94,105,145,146,147] will also likely contribute to the ability of the cell to strive under osmotically challenging growth conditions. An understanding of the role of ectoines as highly effective microbial osmostress protectants therefore seems rather straightforward. However, it is not clear yet how the thermoprotective effects of ectoines [77,78,79,97,121] are achieved on a biochemical and molecular level. While the portrayal of ectoines as chemical chaperones is suggestive, the molecular mechanisms underpinning the function-preserving characteristics of these compounds do not necessarily need to be the same at high and low growth temperatures. From the perspective of basic science, and with respect to practical applications, studies addressing the function of ectoines as thermolytes might prove to be highly rewarding. 

The core of the ectoine/5-hydroxyectoine biosynthetic route and the properties of the involved enzymes (Figure 2) are now reasonably well understood, but nevertheless require further focused efforts to attain a detailed structure/function description of each of the involved biocatalysts. This has already been accomplished in quite some detail for the ectoine hydroxylase (EctD) through biochemical, structural, site-directed mutagenesis, and modeling approaches [93,177,186]. Ectoine/5-hydroxyectoine producers live in ecophysiological varied and often stressful habitats that might require evolutionary adaptation of the underlying biosynthetic enzymes. The phylogenomic data (Figure 5) might therefore serve as a guide to choosing those ectoine biosynthetic enzymes that are best suited for structural approaches. Our extended overview of the genetic context of *ectC* genes underscored recent reports on the widespread occurrence of solitary EctC-type proteins [92,93]. Their biochemical properties and potential physiological function [221] are so far unresolved and certainly should be a topic for future studies.

From the view of basic science, the understanding of the genetic and physiological regulatory circuits controlling *ect* gene expression in response to environmental and cellular cues is a pressing issue. There is no consistent picture of how this is accomplished, and the literature on this topic is plagued with claims that are not sufficiently substantiated by experimental data. In the bacteria studied so far, transcription of the ectoine/5-hydroxyectoine biosynthetic genes is under osmotic control, fully consistent with the major physiological function of these potent osmostress protectants. However, the underlying genetic regulatory mechanisms might differ in different microbial species and might entail promoters that operate independently of specific regulatory proteins [116], while others might be dependent on such transcription factors (e.g., RpoS; SigB) [77,78]. Most interesting is the association of the *ect* genes in many *Proteobacteria* with a gene (*ectR*) that encodes a member of the MarR super-family of transcriptional regulators (Figure 5). So far, EctR has only been functionally studied in a few aerobic, moderately halophilic methylotrophic bacteria (*M. alcaliphilum* 20Z, *M. alcalica, M. thalassica*), where it serves as a repressor of *ect* gene expression [99,198,215], but does not seem to be critical for their osmostress-responsive transcription [198]. The environmental or cellular cues to which EctR responds in its DNA-binding activity are unknown and hence our understanding of the role of this intriguing regulatory protein in controlling *ect* gene expression is rather incomplete. 

The widespread occurrence of ectoine/5-hydroxyectoine producers in terrestrial and marine habitats (Figure 5) also leads to the presence of cell-free ectoines in natural ecosystems when these cells are osmotically down-shocked or when they lyse [233]. The recovery of ectoines by microorganisms from environmental sources via high affinity transport systems will aid these bacteria in their attempts to withstand osmotic and temperature extremes. These transporters are of ecophysiological importance not only for the acquisition of ectoines, where they act as stress protectants, but they also seem to serve as recycling systems for newly synthesized ectoines that are either leaked or actively excreted from the producer cells [161,304]. Continued efforts are required to understand the physiological relevance and molecular underpinning of this latter process [161,304,305], and to further enhance our understanding of the structure/function relationship of ectoine/5-hydroxyectoine importers [241,261,267,268]. Furthermore, new selection or screening procedures might lead to the identification of additional members (and perhaps also novel types) of transporters for ectoines.

Ectoine/5-hydroxyectoine import systems (e.g., EhuABCD, UehABC) also play a crucial role in scavenging these compounds from scarce environmental sources, where these nitrogen-rich molecules (Figure 1B) are used as nutrients. Consistent with their important contribution to ectoine/5-hydroxyectoine catabolism, the transcription of the *ehu* and *ueh* structural genes is substrate-inducible but is not subjected to osmotic control [265,267,268,270]. In contrast to the already rather well studied ectoine/5-hydroxyectoine biosynthetic genes, an understanding of the biochemistry of the catabolic enzymes is in its infancy. Although experimentally testable proposals for the catabolism of 5-hydroxyectoine and ectoine have been made (Figure 6B) [158,263], the inspection of the corresponding gene clusters not only revealed a considerable variation in their genetic organization but also in their gene content (Figure 6A) [158,263,270]. This variation suggests that alternatives (or additions) to the proposed catabolic pathways might exist in microorganisms. 

Recent studies on the genetics of the transcriptional control of ectoine/5-hydroxyectoine utilization genes paint a rather complex picture of this process [270], but they have already uncovered a central role of the MocR/GabR-type EnuR regulator and its ectoine-derived inducers *N*-α-ADABA and DABA [270,277]. Nevertheless, further in-depth studies are required to elucidate the complete regulatory circuit controlling import and catabolism of ectoines and to illuminate the role played by the feast-and-famine regulator AsnC and the NtrYX two-component regulatory system [270]. Although about 45% of the genomes of the 539 predicted ectoine/5-hydroxyectoine consumers simultaneously possess the EnuR, AsnC, and NtrYX regulatory systems implicated in controlling ectoine/5-hydroxyectoine catabolic genes [270], there is again a considerable variation in their phylogenetic distribution, suggesting that differently configured regulatory circuits control the catabolism of ectoines in different microorganisms. 

The recent discovery of ectoine/5-hydroxyectoine biosynthetic genes in the halophilic protist *H. seosinensis* [100,101] and the salt-stress-responsive production and import of ectoine in *S. salinarum* [102] came at a considerable surprise for scholars of microbial osmostress response systems [104]. The data reported by Harding et al. [100,101] suggest the presence of ectoine/5-hydroxyectoine biosynthetic genes in Eukarya other than *H. seosinensis* and *S. salinarum*. As a case in point, the re-programming and induction of ectoine/5-hydroxyectoine uptake and catabolic genes in the marine bacterium *R. pomeroyi* in a co-culture with the diatom *T. pseudonana* [303] strongly suggest that this eukaryote produces and releases ectoines, because enhanced expression of these genes by *R. pomeroyi* DSS-3 is strictly dependent on ectoine-derived metabolites [270,277]. Taken together, these findings underscore the importance of ectoines not only as effective stress- and cytoprotectants but also suggest an important function of these nitrogen-rich compounds as mediators of ecophysiologically important food webs. Ectoines will remain a fascinating research topic for many years to come, both from the perspective of basic science and applied approaches.

## Figures and Tables

**Table 1:** Analysis of the genetic neighborhood of the 582 EctC-type proteins obtained through genome database analysis.

Gene	*ectC* (in Total)	*ectC* (within *ect* Cluster)	*ectC* (Solitary)	*ectD* (within *ect* Cluster)	*ectD* (Separated from *ect* Cluster)	*ask_ect* (within *ect* Cluster)	*ectR*
Abundance	582	437	145	259	68	133	97
